# Differential roles of cyclin D1 and D3 in pancreatic ductal adenocarcinoma

**DOI:** 10.1186/1476-4598-9-24

**Published:** 2010-02-01

**Authors:** Nikolina Radulovich, Nhu-An Pham, Dan Strumpf, Lisa Leung, Wing Xie, Igor Jurisica, Ming-Sound Tsao

**Affiliations:** 1 Ontario Cancer Institute and Princess Margaret Hospital, University Health Network, Toronto, Ontario, M5G 2M9, Canada; 2 Department of Laboratory Medicine and Pathobiology, University of Toronto, 1 King's College Circle, Toronto, Ontario, M5S 1A8, Canada; 3 Department of Medical Biophysics, Ontario Cancer Institute, Princess Margaret Hospital, 610 University Avenue, Toronto, Ontario, M5G 2M9, Canada; 4 Campbell Family Institute for Cancer Research, PMH, University Health Network, 101 College St., TMDT, Toronto, Ontario M5G 1L7, Canada; 5 Department of Computer Science 6 King's College Rd., University of Toronto, Toronto, Ontario, M5S 1A4, Canada

## Abstract

**Background:**

The cyclin D1 (CCND1) and cyclin D3 (CCND3) are frequently co-overexpressed in pancreatic ductal adenocarcinoma (PDAC). Here we examine their differential roles in PDAC.

**Results:**

CCND1 and CCND3 expression were selectively suppressed by shRNA in PDAC cell lines with expression levels of equal CCND1 and CCND3 (BxPC3), enhanced CCND1 (HPAC) or enhanced CCND3 (PANC1). Suppression of cell proliferation was greater with CCND3 than CCND1 downregulation. CCND3 suppression led to a reduced level of phosphorylated retinoblastoma protein (^Ser795^p-Rb/p110) and resulted in decreased levels of cyclin A mRNA and protein. A global gene expression analysis identified deregulated genes in D1- or D3-cyclin siRNA-treated PANC1 cells. The downregulated gene targets in CCND3 suppressed cells were significantly enriched in cell cycle associated processes (p < 0.005). In contrast, focal adhesion/actin cytoskeleton, MAPK and NF B signaling appeared to characterize the target genes and their interacting proteins in CCND1 suppressed PANC1 cells.

**Conclusions:**

Our results suggest that CCND3 is the primary driver of the cell cycle, in cooperation with CCND1 that integrates extracellular mitogenic signaling. We also present evidence that CCND1 plays a role in tumor cell migration. The results provide novel insights for common and differential targets of CCND1 and CCND3 overexpression during pancreatic duct cell carcinogenesis.

## Background

In normal cells, growth factors and mitogenic signaling stimulate the expression of D-cyclins and E2F activity to drive G0/G1 to S phase cell cycle progression [1]. D-cyclins bind to and activate CDK4/6, which phosphorylate the retinoblastoma tumor suppressor protein (Rb) leading to its inactivation and the release of the E2F transcription factors and expression of genes critical for cell cycle progression. In many human cancers, one or more of these regulators for G1/S cell cycle transition are often altered in their expression or function [2]. The inactivation of the tumor suppressor p16 [3] and the overexpression of cyclin D1 (CCND1) and/or cyclin D3 (CCND3) are common in pancreatic ductal adenocarcinoma (PDAC). During multi-stage pancreatic duct cell carcinogenesis, CCND1 overexpression occurred mainly in late stage pancreatic intraepithelial neoplastic (PanIN) lesions, while CCND3 and cyclin A (CCNA) overexpression occurred earlier and at higher frequencies [4]. In contrast to CCND1 and D3, which are often differentially over-expressed in PDAC [5], CCND2 appears to play a role mainly in the proliferation of pancreatic islet β-cell [6], and its mRNA expression was infrequently detected in PDAC and pancreatic cancer cell lines [5,7]. We hypothesize that in PDAC, CCND1 and CCND3 have different regulatory effects on the Rb/E2F complex leading to the transcription activation of different target genes with global effects on the cell cycle. Previous studies suggest that there are non-redundant roles of D-cyclins by their various combinations that associate with different biological contexts (e.g. embryogenesis, growth and differentiation) as well as in carcinogenesis [8]. Many factors or mechanisms may contribute to the deregulation of D-cyclins in PDAC. These include the enhanced expression of growth factors, including platelet derived growth factor (PDGF), amphiregulin and transforming growth factor (TGF)-α [5]. The induction of CCND1 has been associated with enhanced activities of multiple signaling pathways already implicated in PDAC, including ERBB2/STAT3, NOTCH1/NF-κB and sonic hedgehog [9-11]. It remains unclear whether the transcriptional targets of D-cyclins/Rb/E2F pathway are limited to regulators of the cell cycle or if they also have activities on other pathways in PDAC, including apoptosis, invasion and sensitivity to anti-cancer agents. In this study, we have examined the overlap and divergence of CCND1 and CCND3 targets and putative functions in PDAC cell lines BxPC3, HPAC and PANC1, including their roles in cellular proliferation, senescence, migration and global gene transcription. Levels of CCND1 or CCND3 were suppressed by using shRNA expressing lentivirus in three pancreatic cell lines, BxPC3, HPAC and PANC1, that expressed differential D1/D3-cyclins. Effects on global gene transcript targets using microarray was examined in PANC1 cells transfected with either D1 or D3 cyclins siRNA. The functional annotation, enrichment and relationship of affected genes were identified using three publicly available databases: Gene Ontology (GO), KEGG pathways, and the Interolog Interaction Database (I^2^D), a protein-protein interactions database.

## Materials and methods

### Cell lines and growth/senescence assays

Human pancreatic cancer cell lines, BxPC3, HPAC and PANC1 were obtained from the American Type Culture Collection (Manassas, VA). BxPC3 expressed relatively comparable levels of CCND1 and CCND3. HPAC showed differentially higher expression of CCND1 than CCND3, while PANC1 expressed higher levels of CCND3. Cell proliferation was measured by MTS assay (Promega, Madison, WI) as recommended. The senescence-associated β-galactosidase assay was performed as described previously [12].

### Viral vector constructs

DNA oligonucleotides were generated, annealed and ligated into lentiviral vectors plko.YFP or plko.1puro as described previously [13]. The first set of siRNA sequences (Dharmacon Research Inc., Lafayette, CO), CCND1 (GTTCGTGGCCTCTAAGATGAA; shD1_1) and CCND3 (ACAGAATTGGATACATACACC; shD3_1) were subcloned into pLKO.YFP vectors. The second set of siRNA sequences, CCND1 (CCTGAGGAGCCCCAACAACTT; shD1_2) and CCND3 (TAGATGGCTCCTCTCAGTACT; shD3_2) were subcloned into the pLKO.puro vector. A control shRNA sequence was adopted from a non-silencing control siRNA sequence (TTCTCCGAACGTGTCACGT) (Qiagen Inc., Mississauga, ON). Lentiviruses preparation and subsequent transduction was performed as described previously [14]. Vector expressing CCND1, pBMN-CCND1, was constructed by inserting full-length CCND1 cDNA (*Bam*HI/*Xho*I) into pBMN-I-GFP (Garry Nolan laboratory, Stanford University, CA).

### Western blot analysis

Immunoblotting was performed using whole protein extracts from exponentially dividing cells, and probed with the following antibodies against cyclin D3 (BD Biosciences, St. Jose, CA), cyclin D1 (BD Biosciences), Rb (Cell Signalling Technology (CST)), p-Rb (Ser780, Ser795 and Ser807/811, CST), cyclin A (BD Biosciences), Cdk4 (CST), Cdk6 (CST), Erk (CST), p-Erk (Thr202/Tyr204; CST), Akt (CST), p-Akt (Ser473; CST), GAPDH (Abcam), and secondary mouse or rabbit conjugated IgG-horseradish peroxidase (CST). Each blot was repeated at least three times. Densitometry was performed using the ImageJ software to measure the integrated intensity and band size. Levels were calculated relative to the GAPDH standard control on each blot.

### Transwell migration and invasion assays

Trypsin dissociated cells (5 × 10^4^) were resuspended in medium containing 2% bovine serum albumin and added on to the top chambers of 24-well Transwell plates coated with 7.5 μg collagen type IV (CN IV) for migration assay and 5 μg/mL fibronectin containing medium was added to the bottom chambers. Cells were incubated for 48 hours at 37°C in a humidified incubator, then fixed with 0.1% glutaraldehyde-PBS for 20 minutes, rinsed with double-distilled water, and stained with 0.2% crystal violet for an hour. Filters were washed thoroughly with double-distilled water and non-motile cells on top of filters were removed using cotton swabs.

### Microarray transcriptional profiling

Cyclin D dependent gene expression was determined in PANC1 cells transfected with D1- or D3-cyclin siRNA obtained as SMARTpool duplexes, or non-specific siRNA as a control (Dharmacon Products, Thermo Scientific, Lafayette, CO) as described previously [4]. Lentiviral transduced cells were omitted from this assay due to possible transcriptional effects dependent on integration of lentiviral genes [15]. RNA was isolated 72 hours post transfection, and analyzed using an Affymetrix U133 plus 2 microarray (Santa Clara, CA). The raw microarray data were pre-processed using the RMAexpress v0.3 and values were log_2_-transformed [16]. Expression levels from D1- or D3-cyclin siRNA-treated cells were compared to levels from non-specific siRNA control cells, and filtered to include probe sets that were altered at least 2-fold. Probe sets were annotated against UniGene (build Hs.197) using BLASTN [17] or GeneAnnot tool http://bioinfo2.weizmann.ac.il/geneannot/[18]. Annotated probe sets were then matched with an Entrez Gene ID, Gene Symbol and Gene Name using the Entrez Gene database of 2006-11-28 and SwissProt ID (Build 51.5). Analysis was completed with reference to the altered genes rather than the probe sets, herein termed "target genes".

### Reverse transcription and quantitative PCR (RT-QPCR)

RT-QPCR was used to validate microarray results of a subgroup of gene targets. RT was completed at 42°C with SuperScript II RNase H^- ^reverse transcription kit (Invitrogen, Burlington, ON, Canada). QPCR was performed with 10 ng of the first-strand cDNA synthesis mixture as a template and individual primer sets [See Additional file 1] using a Stratagene MX3000P instrument (La Jolla, CA). Primers were designed using publicly available primer bank [19].

Gene expression levels in the samples were calculated relative to control using the comparative C_T _method [20]: ΔΔC_T _= ΔC_T sample _- ΔC_T control_, fold change = 2^-ΔΔCT^. The geometric mean of ribosomal protein S13, β-actin, TATA binding protein and β-2-microglobulin were used to normalize the expression data (ΔC_T_).

### Functional annotation of gene expression data

To systematically annotate and predict biological processes and pathways of target genes affected by the suppression of cyclin D levels we employed three strategies: 1) GO term annotation and enrichment analysis using GoMiner (http://discover.nci.nih.gov/gominer/GoCommandWebInterface.jsp, version 2007-06) [21]; 2) KEGG pathways annotation using the selection of 38 human cancer and signaling specific KEGG pathways (ftp://ftp.genome.jp/pub/kegg/; downloaded on 2007-07-30) [22]; 3) target genes matching to protein-protein interactions (PPIs) in I^2^D (Interolog Interaction Database) v1.7 http://ophid.utoronto.ca/i2d[23].

GoMiner was used to determine whether the target genes as well as the corresponding proteins in PPI networks showed enrichment in certain GO biological processes. Enrichment is defined as the proportion of changed genes in the category relative to the expected proportion for the entire microarray. Significance was tested using one-sided Fisher's exact test and the false-discovery rate (FDR) threshold was set at 0.05, with 1000 randomizations [24].

Additional functional annotation was performed using a selection of 38 biological signaling and disease-related KEGG pathways. To assess enrichment for specific KEGG pathways representation in the target genes the proportion of target genes was compared with proportion of genes expected from the entire gene set on U133 Plus 2.0 microarray using Fisher's exact test. Obtained p-values were corrected for multiple testing of the 38 KEGG pathways.

Experimental PPI networks were generated by querying the I^2^D database with the target genes to obtain corresponding proteins and their immediate interacting partners (i.e., depth of 1). Relationships between the interacting proteins were added to the same network (depth of 1 plus), resulting in PPI networks with 576 proteins and 4557 interactions for CCND3 uniquely deregulated genes, 1362 proteins and 12135 interactions for CCND1 uniquely deregulated genes, and 289 proteins and 1763 interactions for common genes deregulated in both CCND1/CCND3 siRNA, used in subsequent analyses. The proteins in PPI networks were annotated with GO biological processes and tested for enrichment using GoMiner as described above. Significant KEGG pathways were determined for target genes and their interacting proteins in PPI networks by testing their proportions against expected proportions estimated from 1000 randomly-generated PPI networks obtained by querying a subset of I^2^D for genes represented on Affymetrix U133 Plus 2.0 chip, with the same number of proteins as cyclin D1/D3 deregulated target genes matched in I^2^D (81 for cyclin D1 deregulated target genes in PPI, 37 for cyclin D3 and 21 for deregulated target genes in common to cyclin D1/D3). The Student's t-test was then used to compare the proportion in the experimentally determined PPI network against the distributions in random networks, as previously described [25]. Obtained p-values were corrected for multiple testing of the 38 KEGG pathways used for annotation. PPI networks were annotated, visualized and analyzed using NAViGaTOR v2.0 http://ophid.utoronto.ca/navigator/[26].

## Results

### Effects of D-cyclin downregulation on G1-S cell cycle regulators

**Figure 1:**
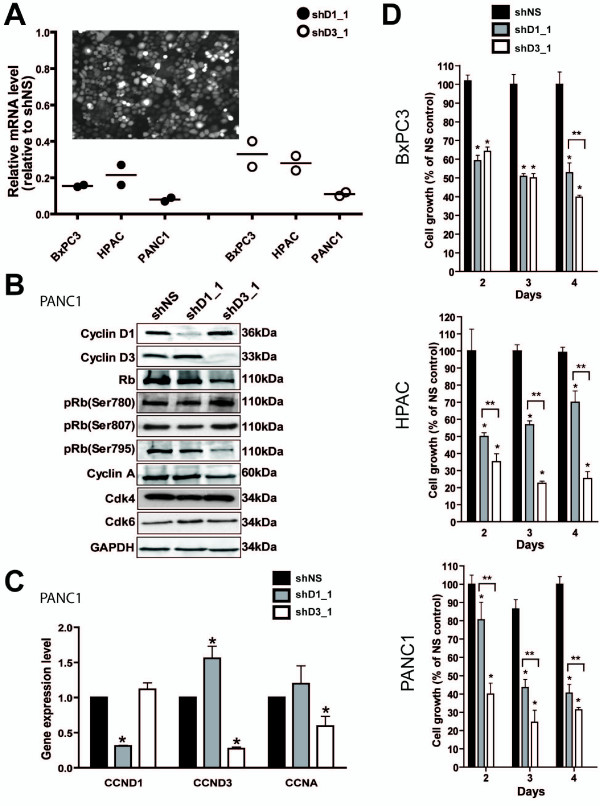
**Cyclin D1/D3 suppression effects on cell cycle and proliferation**. (A) The expression of lentiviral shD1_1 or shD3_1 decreases the mRNA of D1- or D3-cyclin, respectively in BxPC3, HPAC and PANC1 cells. Bars represent medians. The insert is a representative image of shNS_YFP transduced PANC1 cells (insert). (B) Immunoblots show effects of shD1_1, shD3_1 or non-specific shNS on cell cycle-specific proteins D-cyclins, Rb, cyclin A (CCNA) and equal loading control GAPDH in PANC1 cells. (C) Differential effects of shD1_1 or shD3_1 expression on mRNA levels of the D-cyclins and cyclin A in PANC1 cells. (D) Proliferation was decreased in all three cell lines over a period of 4 days after 2 days transduction with shD1_1 or shD3_1 compared with shNS. Values are mean ± standard error. An asterisk (*) designates significant differences between test and control samples (p < 0.001, two-way RM ANOVA and Bonferroni posttests). Significantly different tested pairs are designated by brackets.

The YFP transduction marker was detected at 48 hours in >90% of cells following transduction with lentiviruses containing CCND1 shRNA (shD1_1), CCND3 shRNA (shD3_1) or non-specific shRNA (shNS) in all three pancreatic cancer cell lines BxPC3, HPAC and PANC1 (Figure 1A insert). The transduction using shD1_1 downregulated CCND1 mRNA expression by 79% to 92% and shD3_1 downregulated CCND3 mRNA expression by 67% to 89% in all three cell lines (Figure 1A). Consistent with these effects, CCND1/D3 protein levels were significantly decreased (p < 0.01) as shown in the representative PANC1 cell line (Figure 1B).

CCND1 suppression significantly increased CCND3 RNA and protein levels (Figure 1B and 1C), but CCND3 suppression did not result in a similar compensatory upregulation in the CCND1 levels. Suppression of CCND3 was also associated with decreased Rb and p-Rb(Ser795) levels (p < 0.01), but not pRb(Ser780) or pRb(Ser807). In contrast, suppression of CCND1 resulted in significant decrease in p-Rb(Ser807) levels (p < 0.01), but not pRb(Ser780) or pRb(795) (Figure 1B: Additional File 2). Suppression of CCND3 but not CCND1 decreased the mRNA and protein levels of cyclin A (CCNA), an Rb-E2F transcription target (Figure 1B and 1C). The protein levels of Cdk4/6 remained unchanged in either shD1_1 or shD3_1 relative to shNS treated PANC1 cells (Figure 1B).

### D-cyclin dependent proliferation

**Figure 2:**
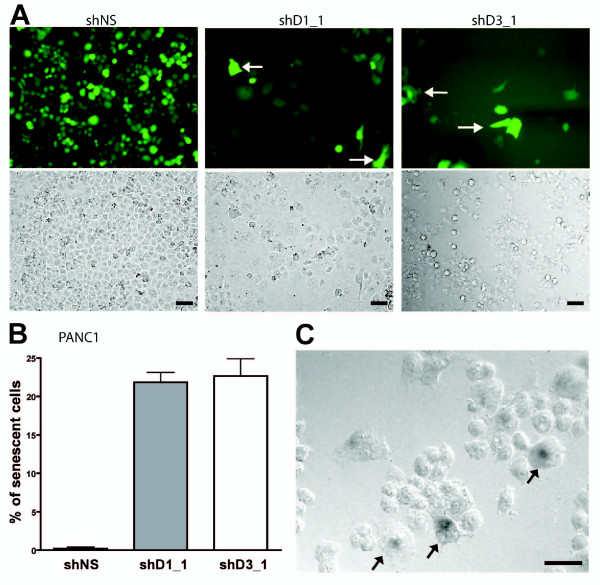
**D-cyclin suppression induces senescence**. (A) Representative images show transduced YFP expressing PANC1 cells, 20 days post-transduction. Arrows indicate YFP cells of large senescent-like morphology that were quantified (B), bars are mean ± standard error. (C) A representative image shows that larger cells of shD3_1 transduced PANC1 display senescence associated β-galactosidase activity (arrows). Scale bars designate 20 μm.

Cell proliferation was similarly suppressed by downregulation of both CCND1 and CCND3 (p < 0.001) in all three cell lines. The shD3_1 achieved a slightly but significantly greater suppression after 4 days in BxPC3 that expresses comparable levels of both D-cyclins (Figure 1D). Suppression of the CCND3 induced a significant greater loss of proliferation (p < 0.001) than CCND1 suppression in both the HPAC cells that express higher CCND1, and in PANC1 cells that express higher CCND3. Therefore, regardless of the basal CCND1/D3 expression levels in the PDAC lines, proliferation was inhibited to a greater extent in cells transduced with shD3_1 compared with shD1_1. This decrease in proliferation was associated with an onset of cellular senescence. A loss of the YFP transduction marker occurred 5 to 20 days post transduction without the induction of apoptosis [see Additional file 3]. At 20 days, YFP-labeled cells were larger and flatter (Figure 2A), and represented approximately 20% of total cell population (Figure 2B). These larger cells stained positive for β-galactosidase, a marker of cellular senescence (Figure 2C). Growth of PANC1 cells *in vivo *resulted in tumor without the YFP marker following subcutaneous injections in SCID mice with shD1_1 or shD3_1 infected cells, suggesting that cells with stable suppression of either D1- or D3-cyclins did not proliferate or survive (data not shown).

To confirm that results with shD1_1 or shD3_1 were specific to shRNA expression and not due to off-target effects, different target sequences of shRNA against the D-cyclins (shD1_2 and shD3_2 in pLKO.puro vectors) were tested in PANC1 cells. Similar results were obtained showing that CCND3 suppressed cells were growth inhibited to a greater extent than the CCND1 suppressed cells [See Additional file 4].

### Global transcriptome changes associated with D-cyclin suppression

Transcriptional profiling was performed on PANC1 cells transfected with siRNA against CCND1, CCND3, or a non-specific control (NS). Our dataset included 189 probe sets corresponding to 165 deregulated genes that were altered at least two-fold compared to the control and associated with CCND1 suppression, and 107 probe sets corresponding to 106 deregulated genes related to CCND3 suppression. The cyclin D1/D3 datasets shared 36 probe sets corresponding to 34 deregulated genes [see Additional file 5, Table S2A]. Probe sets and their corresponding deregulated genes unique to either cyclin D1 or D3 suppression are shown in Additional file 5 (Table S2B and S2C, respectively).

### Common D-cyclin regulated genes

**Figure 3:**
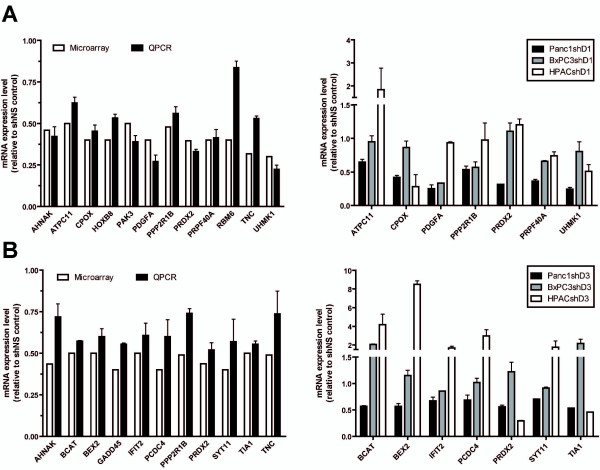
**Levels of gene expression changes were similar using either microarray or quantitative PCR (QPCR) assays**. Comparison was performed on 20 target genes that were downregulated in PANC1 cells treated with (A) CCND1 siRNA or (B) CCND3 siRNA. A subset of genes was selected to determine expression levels in all three cell lines (PANC1, BxPC3 and HPAC) after suppression of CCND1 or CCND3.

D-cyclins co-regulated target genes potentially are genes that were downregulated as a result of either D1- or D3-cyclin suppression (Table 1). Listed genes are from largest to smallest decreases in expression levels unique to CCND1 or CCND3 siRNA treatment, and their shared effects. Changes in gene expression of selected downregulated genes identified by microarray analysis were also similarly observed using RT-QPCR analysis in both CCND1 and CCND3 siRNA treated PANC1 cells (Figure 3A and Figure 3B). These targets were primarily selected as they have also been shown to be deregulated in several publicly available profiling databases of PDAC [27-32]. To determine if these targets are PANC1 cell specific, we also assessed their expression in shD1_1 (Figure 3A) and shD3_1 (Figure 3B) treated BxPC3 and HPAC lines. The expression levels of selected genes are more similar between PANC1 and BxPC3 cells than to HPAC when either D1- or D3-cyclin was suppressed.

**Table 1:** List of downregulated genes in PANC1 cells transfected with cyclin D1 or cyclin D3 siRNA.

Cyclin D1 down regulated genes			Cyclin D3 down regulated genes			Common Cyclin D1 and D3 down regulated genes			
**Fold change with D1 siRNA**	**Gene Symbol**	**UniGene**	**Fold change with D3 siRNA**	**Gene Symbol**	**UniGene**	**Fold change with D1 siRNA**	**Fold change with D3 siRNA**	**Gene Symbol**	**UniGene**

0.1626	USP24	Hs.477009	0.3528	DHX40	Hs.29403	0.105	0.4495	SLC18A2	Hs.369009
0.2594	UHMK1	Hs.127310	0.3879	FNDC3B	Hs.159430	0.3169	0.4886	TncRNA	Hs.523789
0.2817	PDZD8	Hs.501149	0.3888	GPR180	Hs.439363	0.3967	0.4357	PRDX2	Hs.432121
0.3076	HIPK3	Hs.201918	0.3888	LOC144874	Hs.439363	0.4124	0.4593	LOC400043	Hs.19193
0.336	MALAT1	Hs.642877	0.3939	SLC39A6	Hs.79136	0.4233	0.4004	ELOVL6	Hs.412939
0.3422	GPR137B	Hs.498160	0.405	C1orf52	Hs.26226	0.4569	0.4731	PTBP2	Hs.591430
0.3425	SIKE	Hs.632428	0.4093	LIMS3	Hs.535619	0.4592	0.4352	AHNAK	Hs.502756
0.3558	LIN28B	Hs.23616	0.4093	LOC440895	Hs.535619	0.4615	0.4785	LOC348840	Hs.512227
0.3661	MLLT11	Hs.75823	0.4205	C14orf138	Hs.558541	0.4795	0.484	FAM70A	Hs.437563
0.3673	GALNT7	Hs.127407	0.421	CYP26B1	Hs.91546	0.4798	0.4904	PPP2R1B	Hs.584790
0.3739	PRMT2	Hs.154163	0.4223	GADD45B	Hs.110571	0.4901	0.4935	CA5BL	Hs.532326
0.3742	ARL6IP6	Hs.516468	0.4243	DBF4	Hs.485380	0.5116	0.4906	C9orf5	Hs.621479
0.3933	CNOT6	Hs.157606	0.4287	SYT11	Hs.32984				
0.3959	ATG12	Hs.264482	0.4288	SLC35F3	Hs.158748				
0.4142	WDR17	Hs.532056	0.4293	CRLF3	Hs.370168				
0.4191	PAQR3	Hs.632591	0.4334	PPP2CB	Hs.491440				
0.4208	PDGFA	Hs.645488	0.4361	RAB27A	Hs.493512				
0.4296	LRRC58	Hs.518084	0.4433	TMEM140	Hs.567530				
0.4305	SCML1	Hs.109655	0.446	PDCD4	Hs.232543				
0.4315	RBM6	Hs.596224	0.4534	HYMAI	Hs.444975				
0.4405	FAM55C	Hs.130195	0.4534	PLAGL1	Hs.444975				
0.4416	CPOX	Hs.476982	0.4537	ARMC8	Hs.266826				
0.4431	PGM2	Hs.23363	0.4558	IFIH1	Hs.163173				
0.446	PRPF40A	Hs.591637	0.4585	SKP2	Hs.23348				
0.4463	CMTM4	Hs.643961	0.4601	AFG3L1	Hs.534773				
0.4467	FBXO27	Hs.187461	0.4607	BEX2	Hs.398989				
0.4484	HOXB8	Hs.514292	0.4612	ARL6IP5	Hs.518060				
0.4548	DLD	Hs.131711	0.4614	MBOAT1	Hs.377830				
0.4554	LOC205251	Hs.128499	0.4685	SLC39A6	Hs.79136				
0.4578	LGALS8	Hs.4082	0.4731	MAP9	Hs.61271				
0.4622	E2F7	Hs.416375	0.474	GPRC5B	Hs.148685				
0.4644	FAM116A	Hs.91085	0.4786	GABARAPL3	Hs.592014				
0.4668	HRB	Hs.591619	0.4798	C8orf44	Hs.545401				
0.468	PXK	Hs.190544	0.4798	PTTG3	Hs.545401				
0.4686	CHPT1	Hs.293077	0.4798	SGK3	Hs.545401				
0.4712	USP45	Hs.143410	0.486	CDKN1A	Hs.370771				
0.4718	EIF1AX	Hs.522590	0.4873	SERBP1	Hs.530412				
0.4746	FDFT1	Hs.593928	0.4902	IQCK	Hs.460217				
0.4762	C10orf32	Hs.34492	0.4929	DLL1	Hs.379912				
0.478	ETNK1	Hs.29464	0.4948	KDELC1	Hs.408629				
0.4823	UBE2Q1	Hs.607928	0.4959	C12orf57	Hs.591045				
0.4898	FRAT2	Hs.140720	0.4967	IFIT2	Hs.437609				
0.4918	TMEM97	Hs.199695	0.4969	BCAT1	Hs.438993				
0.4926	EML4	Hs.593614	0.4973	CUL3	Hs.372286				
0.4938	ATP11C	Hs.88252	0.4992	ANP32E	Hs.603000				
0.4979	TMSL8	Hs.56145	0.4998	TIA1	Hs.516075				
0.4992	PAK3	Hs.390616							

Dowregulated gene targets in D-cyclin suppressed cells, unique to cyclin D1, cyclin D3, and shared targets of either D1- or D3-cyclin suppression. Genes are listed in order of ascending fold change.

To explore the potential biological functions of the target genes/proteins, annotations are derived from GO biological processes, KEGG pathways and relevant protein-protein interaction (PPI) networks from I^2^D [See Additional file 6]. Potential functions of the shared 34 deregulated cyclin D1/D3 target genes and their interacting proteins are evident from GO biological processes analysis where a significant enrichment (p-value < 0.05) was found in a diverse set of biological processes, including Wnt signaling, and processes involving membrane and vesicle biogenesis and transport [see Additional file 7, Table S4A].

### Cyclin-D3-regulated genes

**Figure 4:**
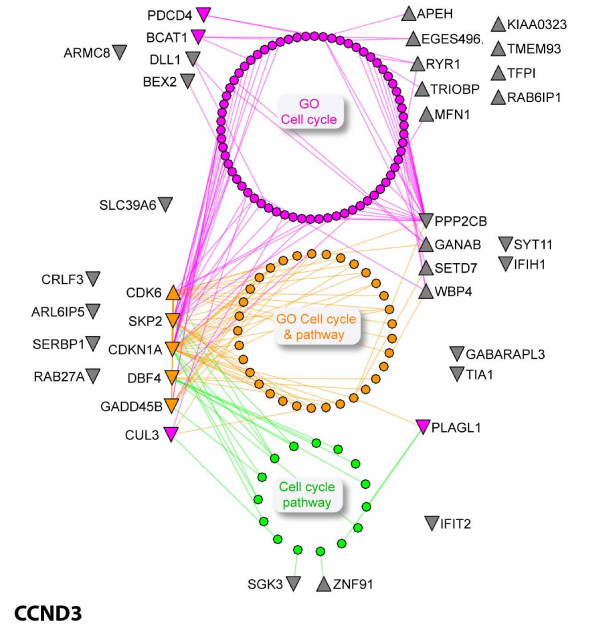
**Cyclin D3-specific deregulated genes and their functional networks**. A protein-protein interaction (PPI) network shows proteins corresponding to deregulated genes in CCND3 siRNA-treated cells and their interacting proteins obtained from I^2^D protein interaction database ver. 1.7 [23] annotated to the cell cycle process. Target genes/proteins whose expression is up-regulated or down-regulated (up/downward triangle nodes, respectively) by at least 2-fold compared with control and their interacting proteins (round nodes) are annotated by either GO cell cycle term (pink nodes, GO:0007049), KEGG cell cycle pathway (green nodes, KEGG hsa4110), common to both annotations (orange nodes), or none of the above (grey nodes). Target nodes positions reflect their interactions with cell cycle annotated nodes (colored), connecting directly to the annotated nodes or via intermediate node(s). Nodes and edges not directly connecting target nodes to pathway-annotated interacting nodes were faded out to reduce network complexity. This network incorporates 37/72 deregulated genes from CCND3 siRNA-treated cells; 36 proteins form a single connected network. A single target IFIT2 was identified in I^2^D, but did not connect to the other nodes at the depth of the presented network. PPI network analysis was done using NAViGaTOR 1.13 ([26]; http://ophid.utoronto.ca/navigator).

CCND3-specific downregulated target genes annotated to GO biological processes were significantly enriched for cell cycle (9/24 genes, p-value ≤ 0.004) and programmed cell death/cell death processes (8/24 genes, p-value ≤ 0.004) [see Additional file 7, Table S4B]. These functions were enriched approximately 6-fold compared to the expected proportion of genes annotated for those functions in the gene population represented on the entire microarray. Five genes were commonly downregulated in both processes, CDKN1A, CUL3, GADD45B, PDCD4 and PLAGL1. Downregulated genes BCAT1, DBF4, MAP9 and SKP2 were specific for cell cycle function, while TIA1, RAB27A and IFIH1 were specific for programmed cell death function. The gene targets and their immediate interacting proteins in PPI networks were also consistently annotated by the cell cycle pathway (Figure 4), which included factors unique and common to GO terms [see Additional file 7, Table S4C] and KEGG pathways [see Additional file 8]. Further, the deregulated target genes and their interacting proteins are also associated with Notch (p-value = 0.0011), induction of apoptosis (p-value = 0.0024) [see Additional file 7, Table S4C] and p53 (p-value = 0.0008) [see Additional file 8] pathways.

### Cyclin D1 regulated genes

CCND1-specific deregulated target genes did not show significant enrichment of particular biological processes as determined by GO terms and KEGG pathways (p-value > 0.05). However, the analysis of the deregulated target genes as well as their interacting proteins from PPI networks did show that CCND1 suppression deregulated potentially cell cycle and cell death processes [see Additional file 7, Table S4D]. Additional functions that were revealed in this analysis included actin cytoskeleton organization and NF-κB cascade as annotated by GO biological processes [see Additional file 7, Table S4D], and focal adhesion and MAPK factors as annotated by KEGG pathways [see Additional file 8], suggesting that CCND1-specific processes are linked closer to external stimuli and signal transduction.

**Figure 5:**
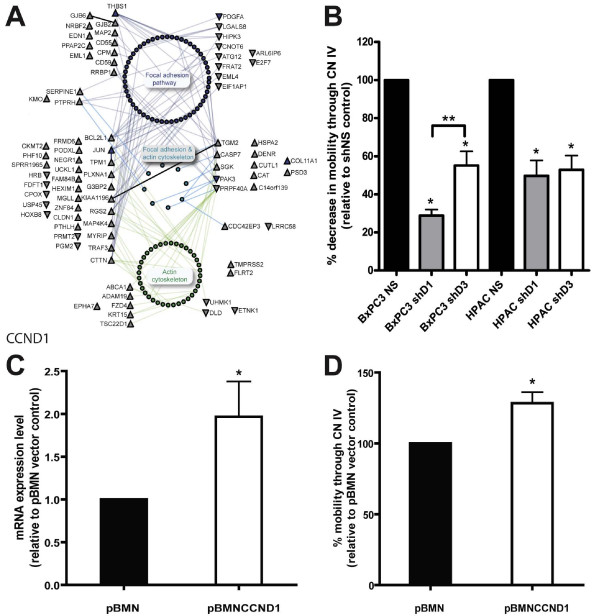
**Cyclin D1-specific deregulated genes are connected to focal adhesion and actin skeleton network**. (A) The PPI network shows 81 proteins corresponding to deregulated genes from CCND1 siRNA-treated cells by at least 2-fold compared with control and their interacting proteins obtained from I^2^D database constitute a single connected network with 79 proteins. Target nodes positions reflect their interactions with pathway-annotated nodes (colored), connecting directly to the annotated nodes or via intermediate node(s). Nodes and edges not directly connecting target nodes to pathway-annotated interacting nodes were faded out to reduce network complexity. Target genes/proteins whose expression is up-regulated or down-regulated (up/downward triangle nodes, respectively) and their interacting proteins (round nodes) are annotated by focal adhesion pathway (blue nodes, KEGG hsa4510), actin cytoskeleton organization and biogenesis (green nodes, GO:0030036), common proteins to both annotations (turquoise nodes), or none of the above (grey nodes). Two proteins, FLRT2 and TMPRSS2, are identified in I^2^D, but did not connect to the other nodes at depth of the presented network. (B) Collagen type IV migration is decreased in BxPC3 and HPAC cells stably expressing shD1_1 compared with control shNS. Representative plots are shown of two repeated experiments completed in duplicate. A significant difference, p < 0.05, in migration between treatments shNS and shD1 or shD3 for each cell line is indicated with an asterisk (*), or between a pair of treatments was indicated with a bracket and asterisks. (C) mRNA levels of CCND1 in PANC1 cells transfected with pBMN compared to pBMN_CCND1 vector are significantly increased, p < 0.05 (*). (D) Collagen type IV migration is significantly increased, p < 0.05, in PANC1 cells upon transient overexpression of CCND1. PPI network analysis was done using NAViGaTOR 1.13 ([26]; http://ophid.utoronto.ca/navigator).

Deregulated focal adhesion and actin cytoskeleton targets and interacting proteins that are unique and common to GO and KEGG annotations in CCND1-supressed cells are mapped in a PPI network (Figure 5A). To test if cyclin D1 has a function in this network, we assessed the effects of shD1_1 and shD3_1 on the migration activity of BxPC3 and HPAC cells through the extracellular matrix component collagen type IV coated membranes. PANC1 was excluded from this analysis due to its very low mobility through collagen (data not shown). Suppression of CCND1 significantly decreased migration activity in BxPC3 (28 ± 3%) and HPAC (49 ± 8%) cells relative to shNS controls (p < 0.05; Figure 5B). The suppression of CCND3 also led to a significant decrease in migration activity relative to shNS in BxPC3 (55 ± 7%, p < 0.05; Figure 5B), however this effect was significantly lower than the effect of cyclin D1 knockdown (p < 0.05; Figure 5B). In contrast, while both shD3_1 and shD1_1 suppressed (52 ± 7%) similarly the migration activity of HPAC cells (p < 0.05; Figure 5B), these levels were less than that observed in shD1_1 treated BxPC3. Importantly, the overexpression of CCND1 (Figure 5C) in PANC1 cells significantly increased their migration activity through collagen type IV coated membranes (p < 0.05; Figure 5D).

**Figure 6:**
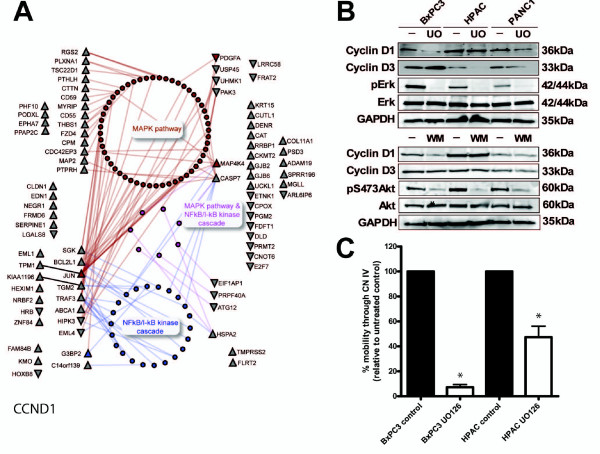
**Cyclin D1-specific deregulated genes are connected to MAPK pathway**. (A) PPI network shows target proteins and their interacting partners annotated by the MAPK pathway (brown nodes, KEGG hsa4010), NFκB/I-κB kinase cascade (blue nodes, GO:0007249), common proteins to both annotations (purple nodes), or none of the above (grey nodes). (B) Immunoblots show responses of cyclin D1/D3 levels to the inhibition of ERK activity with UO126 (UO) for 18 hours, and inhibition of PI3K/Akt activity with wortmannin (WM) for 4 hours. GAPDH levels show equal protein loading. (C) The migration through CN IV of untreated control and UO126-treated BxPC3 and HPAC cells are shown from two repeated experiments completed in duplicate. An asterisk designates a significant difference (p < 0.05) between treated cells and the respective control cells.

Deregulated MAPK and NFκB/I-κB kinase targets and interacting proteins that are unique and common to GO and KEGG annotations in CCND1-suppressed cells are also mapped in a PPI network (Figure 6A). When ERK, a downstream target of the MAPK signaling cascade was inhibited using UO126, a MEK inhibitor, the CCND1 levels were 13 ± 1% in BxPC3 (p < 0.01) and 28 ± 10% in PANC1 cells relative to shNS controls (Figure 6B; Additional File 9). While CCND3 protein levels remained unchanged in UO126 treated BxPC3 and PANC1 cells, the CCND3 levels were 26 ± 7% in UO126 treated HPAC cells (p < 0.01; Figure 6B; Additional File 9) relative to the shNS control. In contrast to ERK, the inhibition of Akt signalling with 60 nM wortmannin (WM) had no significant effects on the protein levels of both D1 and D3-cyclins in all three cell lines.

To test if Erk signaling has a role in cell migration of PDAC cells we assessed the migration of BxPC3 and HPAC cells through collagen in the presence of MEK inhibitor UO126. Compared to the untreated control, UO126 inhibited the migration of BxPC3 and HPAC cells through collagen by 93% and 53% respectively (p < 0.01; Figure 6C).

## Discussion

The differential combination of the three D-cyclins (D1, D2 and D3) suggests that they could function in a cooperative, redundant or distinct manner depending on the biological context [33]. Our results showed that the loss in expression of cyclin D1/D3 inhibited proliferation and resulted in a decrease of the hyperphosphorylated/inactivated Rb in PDAC cells, consistent with the existing concept that D-cyclins drive proliferation in cancer cells [34]. We further demonstrated that D1/D3-cyclins have additional roles, which may cooperate to enhance malignant progression in pancreatic duct cell carcinogenesis.

Inhibition of cell proliferation was greater in CCND3 than CCND1 downregulated cells immediately after shRNA transduction in HPAC and PANC1 cells, and also within 4 days after transduction into BxPC3, irrespective of the differences in basal CCND3 and CCND1 gene expression levels. An upregulation of CCND3 expression in CCND1 suppressed cells suggests a compensatory mechanism possibly attributing to the lesser effect of CCND1 knockdown on PANC1 cell proliferation. This is consistent with previous report that CCND3 partially compensates for loss of CCND2 in mouse B-lymphocytes by Rb phosphorylation on Cdk4 specific sites [35] Additionally, the remaining D-cyclins can increase when a tissue-specific D-cyclin is eliminated during early murine embryonic development [36]. However, our results demonstrate that such compensatory mechanism might be CCND1 specific, as it was absent in CCND3-suppressed PANC1 cells.

The immediate response of CCND3 suppression involved the significant loss of Rb hyperphosphorylation and a putative gain of Rb function. Given that cyclin D partners, Cdk4 and Cdk6, remain unchanged as a result of either CCND1 or CCND3 knockdowns, it is likely that CCND1 and/or CCND3 are rate limiting for Cdk activity in PANC1 cells. Decreases in phosphorylated-Rb has been associated with the knockdowns of CCND1 [37,38], specifically the loss of phosphorylation at Ser780 on Rb has been associated with CCND1/CCND3 proteolysis and growth arrest in cancer cells [39]. We have demonstrated that CCND3-specific loss of phosphorylation at Ser795 on Rb led to a decrease in cyclin A expression, suggesting an effective loss of E2F transcription activity. Due to an overlap in the activity of D-cyclins-cdk4/6 complexes on the 16 putative phosphorylation sites of Rb, specific effects of phospho-Rb profiles on physiological outcome remains unclear [40]. Evidence from the study of diverse cell types shows that patterns of Rb phosphorylation [41] and the resulting effects on cell cycle are associated with selective D-type cyclin response to different mitogenic modes regulating cell growth (mass accumulation) [42], proliferation [43], and cell differentiation [41]. Cyclin D1 expression, the most studied D-cyclin in cancer cell has been associated with anchorage-independent growth, tumorigenicity, angiogenesis, hypoxia response and resistance to chemotherapeutic agents [44]. Our data suggest that CCND1 and CCND3 associate with unique Rb phosphorylation patterns to mediate a differential effect on global gene transcription in pancreatic cancer cells.

The number of deregulated cell cycle genes increases progressively during multi-stage duct cell carcinogenesis. The inactivation of p16^*INK4A *^has been reported in virtually all PDACs [45]. As the main function of p16 is to inhibit the interaction/activity of CCND1 and CDK4/6, a loss of p16 would result in unrestrained activation of the latter, constitutive phosphorylation of Rb and activation of E2F transcription factor. Nevertheless, we and others have reported that despite the putatively ubiquitous loss of p16, CCND1 expression is elevated in 30-50% of PDAC and specifically, we showed that CCND3 is elevated in almost all PDAC [4,46]. Our present results provide important novel insight suggesting that despite the inactivation of p16, CCND3 plays a major role in driving cell cycle progression in PDAC, equal to or even greater than CCND1. This difference on cell cycle effects between CCND1 and CCND3 was also revealed by our PPI network analysis, which shows that CCND3 regulated genes interact predominantly with cell cycle regulatory genes.

We have also demonstrated that prolonged downregulation of CCND1 or CCND3 in PANC1 cells induced cellular senescence. The cellular senescence can be triggered in response to a variety of stresses activated mainly by the p53 and Rb pathways [47]. Mutation of p53 and homozygous deletion of p16^INK4a ^result in PANC1 cells relying on the Rb/cyclinD/Cdk4 pathway for the cell cycle control. Given that the destabilization of CCND1 and CCND3 is a necessary step in induction of growth arrest in PDAC cells [48] it is possible that the inactivation of cyclin D proteins in PANC1 cells resulted in a protracted hyperphosphorylation of Rb and binding to E2F family proteins, eventually leading to cellular senescence.

The results of our microarray and PPI analysis suggest that genes of non-cell cycle pathways, including focal adhesion, MAPK and NFκB are regulated potentially by CCND1. We have shown that cell migration through collagen type IV was significantly inhibited with CCND1 suppression in BxPC3 and HPAC cells. Furthermore, PANC1 migration was increased following overexpression of CCND1. These results suggest that the focal adhesion pathway and actin cytoskeleton are regulated in part by CCND1 in PDAC cells. Correlation between CCND1 overexpression and cellular migration was demonstrated previously in *cyclin D1^-/- ^*MEFS [49]. While our work was under revisions, another group demonstrated that a reduction in CCND1 protein results in decreased invasiveness of PDAC cells further strengthening the findings of the current study [50].

We have identified several CCND1 targets with a role in cell adhesion, including platelet-derived growth factor alpha (PDGFA) whose mRNA expression was affected in two PDAC cells lines with downregulated CCND1 levels. Recent report suggested that PDGFA overexpression in PDA patients correlates with lower survival rate [51]. In addition, Imatinib mesylate (STI571), an inhibitor of alpha- and beta-platelet-derived growth factor receptors (PDGFR) has recently been tested in clinical trials against pancreatic cancer [52]. The relationship between CCND1 and PDGFA signalling pathway warrants further examination, especially for their possible cooperative mechanisms to promote the invasiveness of PDAC cells.

Although to a lesser extent than CCND1, CCND3 knockdown also resulted in decreased migration through collagen type IV in BxPC3 and HPAC cell lines suggesting that D type cyclins might have overlapping roles in cellular migration. The extent of CCND1/CCND3 effects on cellular migration appears to be cell type specific.

Decreases in CCND1 levels in response to MAPK or Akt inhibition confirmed a stronger response of CCND1 to the receptor tyrosine kinase (RTK) signaling pathways in PDAC than CCND3. Although current evidence suggests primarily that mitogenic signaling regulates D-cyclins in a unidirectional pathway to activate E2F-dependent activation of cell cycle progression, consistent with our findings, Ginsberg et al. demonstrated a possible feedback loop whereupon E2F gene targets include effectors of the MAPK and Akt pathway in osteosarcoma cells [53,54]. We observed that selective E2F gene targets depend on specific D-type cyclin regulation.

## Conclusions

In this manuscript we show that the loss of cell proliferation in PDAC cell lines is more pronounced following cyclin D3 suppression compared to that of cyclin D1. While cyclin D3 associated gene expression was enriched for cell cycle processes, cyclin D1 associated expression changes showed greater association with focal adhesion/actin cytoskeleton, MAPK and NFκB signaling. We demonstrated that cyclin D1 has a role in promotion of cell mobility which is consistent with cyclin D1 occurring mainly in late stages of pancreatic intraepithelial neoplastic progression, despite the early ubiquitous inactivation of p16.

## Competing interests

The authors declare that they have no competing interests.

## Authors' contributions

NR designed and carried out the molecular studies, participated in the gene expression data analysis and drafted the manuscript. NP participated in the data analysis and helped to draft the manuscript. DS performed gene expression and protein-protein interaction networks data analysis, and helped to draft the manuscript. LL carried out the validation experiments of microarray data analysis and WX performed protein-protein interaction networks data analysis. IJ directed overall data analysis and participated in network analysis. MT conceived and provided overall supervision of the study. All the authors have read and approved the final manuscript.

## Supplementary Material

Additional file 1 **Supplementary Table 1**. Primers for Real-time PCR of selected genes downregulated by CCND1 or CCND3 siRNA treatment Click here for file

Additional file 2 **Supplementary Figure 1. Quantification of western blots in Figure **1. Values are means ± SEM of at least three Relative Intensities compared to GAPDH standard control in each blot. An asterisk represents statistically significant value calculated by Student t test (P < 0.01). Click here for file

Additional file 3 **Supplementary Figure 2. Pancreatic cancer cell lines with suppressed D-cyclins were rapidly lost in cell culture**. (A) Proportions of YFP-labeled cyclin D1 (shD1_1) or cyclin D3 (shD3_1) suppressed BxPC3, HPAC and PANC1 cells were measured over 20 days compared with control vector (shNS) transduced cells. Asterisks designate significant differences between test samples and control (p-value < 0.001; two-way RM ANOVA and Bonferroni posttests). Double asterisks designate significant differences between pairs (brackets). (B) Cell viability was measured by a presence of mitochondrial membrane potential labeled with DiIC1(5) and exclusion of propidium iodide 5 days post transduction, using a flow cytometry-based assay. Click here for file

Additional file 4 **Supplementary Figure 3. D-cyclin shRNA stable expression in PANC1 cells**. (A) Western blots show decreased levels of cyclin D1 and cyclin D3 as a result of shD1_2 and shD3_2 shRNA expression, respectively. (B) Growth curves for the PANC1 cells were determined by cell counts at day 0, 3, 4, 5 and 6. Values are mean ± SD cell number. Click here for file

Additional file 5 **Supplementary Table 2A-2C. List of downregulated and upregulated genes in common with both D1 and D3 cyclin knockdown**. Probe sets listed are altered at least 2-fold downregulated (green) or upregulated (red) compared with the non-specific siRNA experiment in control cells, and were mapped to UniGene, Entrez Gene and SwissProt. Genes/proteins that were matched to protein-protein interactions (PPIs) in I^2^D ver. 1.7 appear in boldface in SwissProt ID Click here for file

Additional file 6 **Supplementary Table 3. The numbers of genes (all targets) uniquely and commonly altered in D1- or D3-cyclin suppressed PANC1 cells**. The numbers of genes (all targets) uniquely or commonly altered in D1- or D3-cyclin suppressed PANC1 cells. Values are the number of genes functionally annotated in each of the three resources (PPI networks, GO and KEGG). Up and down deregulated gene expressions are common or unique to a D-type cyclin siRNA. The number of functionally annotated genes is increased when targets as well as their immediate interacting proteins are considered. Not applicable (NA). Click here for file

Additional file 7 **Supplementary Table 4A-4D. Functional annotation of deregulated common target genes in D1- or D3-cyclin suppressed cells and their interacting proteins obtained from protein interaction database I^2^D ver. 1.7.**. Significant associations with GO biological processes are shown for common deregulated genes in either D1 or D3 cyclin siRNA-treated cells. Listed are terms where values of enrichment >2 and p-value < 0.05. Enrichment and FDR are as per GoMiner analysis (GO database version 2007-06) described in Materials and Methods. Click here for file

Additional file 8 **Supplementary Table 5. KEGG functional annotation of deregulated target genes in D1- or D3-cyclin suppressed cells and their interacting proteins obtained from protein interaction database I^2^D ver. 1.7**. ([23]; http://ophid.utoronto.ca/i2d). Listed are number of genes mapped to a selection of 38 cancer and signaling KEGG pathways; common target genes are downregulated (down) or upregulated (up) by either D1- or D3-cyclin siRNA treatment (D1/D3). Following are deregulated genes (down or up) unique to either cyclin D1 (D1) or cyclin D3 (D3) siRNA treatment. A significant enrichment (red highlighted) for KEGG pathway cell cycle proteins was found for cyclin D3 downregulated gene targets (p = 0.0048). PPI network analysis showed significant enrichments (red highlighted) of two KEGG pathways: p53 signaling (p = 0.0008) and cell cycle (p = 0.004). Click here for file

Additional file 9 **Supplementary Figure 4. Quantification of western blots in Figure **6. Values are means ± SEM of at least three relative intensities compared to untreated control in each blot. An asterisk represents statistically significant value calculated by Student t-test (P < 0.01). Click here for file
