# Supplementary material for: Differential roles of cyclin D1 and D3 in pancreatic ductal adenocarcinoma
Source: Mol Cancer. 2010 Feb 1;9:24. doi: 10.1186/1476-4598-9-24 (PMC2824633; doi:10.1186/1476-4598-9-24)
Supplement: Additional file 1 — Supplementary Table 1. Primers for Real-time PCR of selected genes downregulated by CCND1 or CCND3 siRNA treatment [file 1476-4598-9-24-S1.DOC]

Supplementary Table1. Primers for Real-time PCR of selected genes downregulated by CCND1 or CCND3 siRNA treatment.
