# Supplementary material for: Differential roles of cyclin D1 and D3 in pancreatic ductal adenocarcinoma
Source: Mol Cancer. 2010 Feb 1;9:24. doi: 10.1186/1476-4598-9-24 (PMC2824633; doi:10.1186/1476-4598-9-24)
Supplement: Additional file 2 — Supplementary Figure 1. Quantification of western blots in Figure 1. Values are means ± SEM of at least three Relative Intensities compared to GAPDH standard control in each blot. An asterisk represents statistically significant value calculated by Student t test (P < 0.01). [file 1476-4598-9-24-S2.DOC]

Supplementary Figure 1. Quantification of western blots in Figure 1

|  |  |  |  |
| --- | --- | --- | --- |
| **Antibody** | ***shNS*** | ***shD1*** | ***shD3*** |
| CCND1 | 1.203+0.05564 | 0.5006+0.1017* | 1.318+0.1477 |
| CCND3 | 1.068+0.02651 | 1.637+0.02104 | 0.2028+0.05325* |
| total Rb | 1.340+0.07249 | 1.208+0.03874 | 0.3595+0.09462* |
| pRb(Ser780) | 0.7873+0.1637 | 0.9502+0.04866 | 1.310+0.1412 |
| pRb(Ser795) | 1.716+0.2427 | 0.7192+0.2721 | 0.4586+0.01520* |
| pRb(Ser807) | 1.066+0.04718 | 0.8554+0.02410* | 1.083+0.07987 |
| Cyclin A | 0.9818+0.03669 | 1.253+0.1547 | 0.7381+0.1298* |
| Cdk4 | 0.7267+0.1873 | 0.6246+0.1755 | 0.6421+0.1983 |
| Cdk6 | 0.9089+0.1086 | 1.087+0.07934 | 1.011+0.04082 |

Values are means+SEM of at least three Relative Intensities compared to GAPDH standard control in each blot. * represents statistically significant value calculated by Student t test (P<0.01).
