# Supplementary material for: Differential roles of cyclin D1 and D3 in pancreatic ductal adenocarcinoma
Source: Mol Cancer. 2010 Feb 1;9:24. doi: 10.1186/1476-4598-9-24 (PMC2824633; doi:10.1186/1476-4598-9-24)
Supplement: Additional file 3 — Supplementary Figure 2. Pancreatic cancer cell lines with suppressed D-cyclins were rapidly lost in cell culture. (A) Proportions of YFP-labeled cyclin D1 (shD1_1) or cyclin D3 (shD3_1) suppressed BxPC3, HPAC and PANC1 cells were measured over 20 days compared with control vector (shNS) transduced cells. Asterisks designate significant differences between test samples and control (p-value < 0.001; two-way RM ANOVA and Bonferroni posttests). Double asterisks designate significant differences between pairs (brackets). (B) Cell viability was measured by a presence of mitochondrial membrane potential labeled with DiIC1(5) and exclusion of propidium iodide 5 days post transduction, using a flow cytometry-based assay. [file 1476-4598-9-24-S3.DOC]

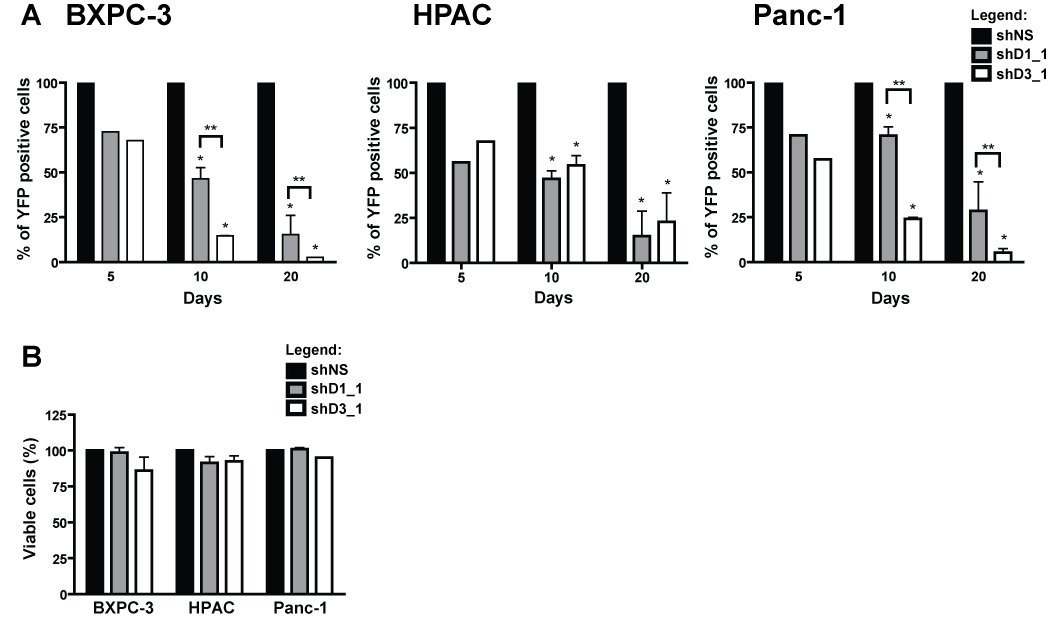


Supplementary Figure 2. Pancreatic cancer cell lines with suppressed D-cyclins were rapidly lost in cell culture. (A) Proportions of YFP-labeled cyclin D1 (shD1_1) or cyclin D3 (shD3_1) suppressed BxPC3, HPAC and PANC1 cells were measured over 20 days compared with control vector (shNS) transduced cells. Asterisks (*) designate significant differences between test samples and control (p-value<0.001; two-way RM ANOVA and Bonferroni posttests). Double asterisks designate significant differences between pairs (brackets). (B) Cell viability was measured by a presence of mitochondrial membrane potential labeled with DiIC1(5) and exclusion of propidium iodide 5 days post transduction, using a flow cytometry-based assay.
