# Supplementary material for: Differential roles of cyclin D1 and D3 in pancreatic ductal adenocarcinoma
Source: Mol Cancer. 2010 Feb 1;9:24. doi: 10.1186/1476-4598-9-24 (PMC2824633; doi:10.1186/1476-4598-9-24)
Supplement: Additional file 4 — Supplementary Figure 3. D-cyclin shRNA stable expression in PANC1 cells. (A) Western blots show decreased levels of cyclin D1 and cyclin D3 as a result of shD1_2 and shD3_2 shRNA expression, respectively. (B) Growth curves for the PANC1 cells were determined by cell counts at day 0, 3, 4, 5 and 6. Values are mean ± SD cell number. [file 1476-4598-9-24-S4.DOC]

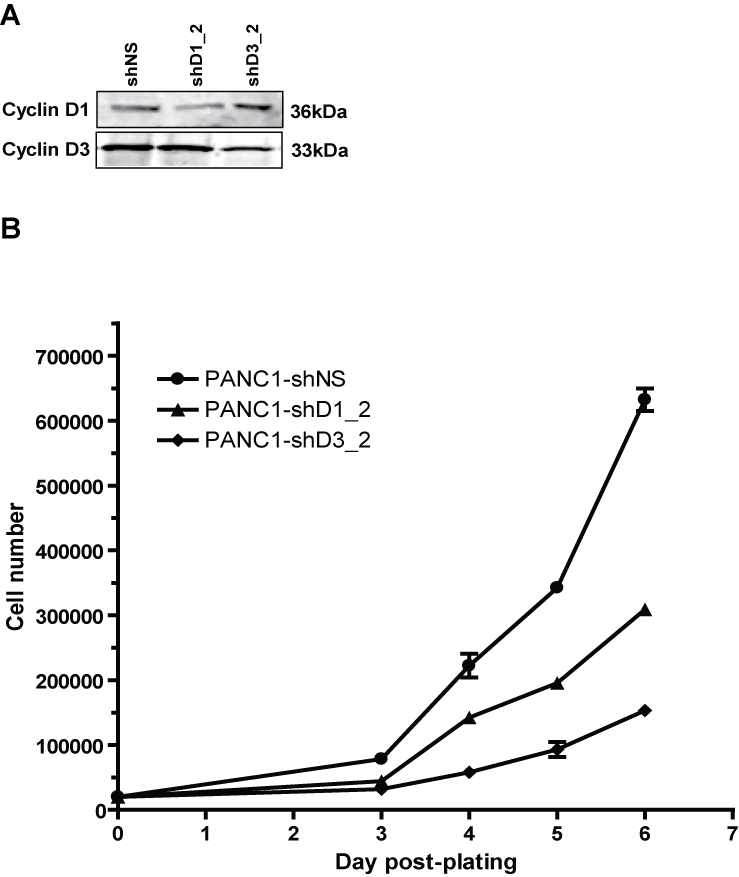


Supplementary Figure 3**.** D-cyclin shRNA stable expression in PANC1 cells. (A) Western blots show decreased levels of cyclin D1 and cyclin D3 as a result of shD1_2 and shD3_2 shRNA expression, respectively. (B) Growth curves for the PANC1 cells were determined by cell counts at day 0, 3, 4, 5 and 6. Values are mean±SD cell number.
