# Supplementary material for: Differential roles of cyclin D1 and D3 in pancreatic ductal adenocarcinoma
Source: Mol Cancer. 2010 Feb 1;9:24. doi: 10.1186/1476-4598-9-24 (PMC2824633; doi:10.1186/1476-4598-9-24)
Supplement: Additional file 5 — Supplementary Table 2A-2C. List of downregulated and upregulated genes in common with both D1 and D3 cyclin knockdown. Probe sets listed are altered at least 2-fold downregulated (green) or upregulated (red) compared with the non-specific siRNA experiment in control cells, and were mapped to UniGene, Entrez Gene and SwissProt. Genes/proteins that were matched to protein-protein interactions (PPIs) in I2D ver. 1.7 appear in boldface in SwissProt ID [file 1476-4598-9-24-S5.DOC]

Supplementary Table 2A. List of downregulated and upregulated genes in common with both D1 and D3 cyclin knockdown

| Probesets | Fold change D1 siRNA | Fold change D3 siRNA | Gene Symbol | Gene Name | Entrez Gene | UniGene | SwissProt |
| --- | --- | --- | --- | --- | --- | --- | --- |
| 220016_at | 0.5 | 0.4 | AHNAK | AHNAK nucleoprotein (desmoyokin) | 79026 | Hs.502756 | **Q09666** |
| 223006_s_at | 0.5 | 0.5 | C9orf5 | chromosome 9 open reading frame 5 | 23731 | Hs.621479 | Q9H330 |
| 239106_at | 0.5 | 0.5 | CA5BL | carbonic anhydrase VB-like | 340591 | Hs.532326 | Q9P170 |
| 204256_at | 0.4 | 0.4 | ELOVL6 | ELOVL family member 6, elongation of long chain fatty acids (FEN1/Elo2, SUR4/Elo3-like, yeast) | 79071 | Hs.412939 | Q9H5J4 |
| 219895_at | 0.5 | 0.5 | FAM70A | family with sequence similarity 70, member A | 55026 | Hs.437563 | Q5JRV8 |
| 242045_at | 0.5 | 0.5 | LOC348840 | hypothetical protein LOC348840 | 348840 | Hs.512227 | Q6ZU74 |
| 226582_at | 0.4 | 0.5 | LOC400043 | hypothetical gene supported by BC009385 | 400043 | Hs.19193 |  |
| 202883_s_at | 0.5 | 0.5 | PPP2R1B | protein phosphatase 2 (formerly 2A), regulatory subunit A (PR 65), beta isoform | 5519 | Hs.584790 | **P30154** |
| 39729_at | 0.4 | 0.4 | PRDX2 | peroxiredoxin 2 | 7001 | Hs.432121 | **P32119** |
| 218683_at | 0.5 | 0.5 | PTBP2 | polypyrimidine tract binding protein 2 | 58155 | Hs.591430 | **Q9UKA9** |
| 230416_at | 0.1 | 0.4 | SLC18A2 | solute carrier family 18 (vesicular monoamine), member 2 | 6571 | Hs.369009 | **Q05940** |
| 227062_at | 0.3 | 0.5 | TncRNA | trophoblast-derived noncoding RNA | 283131 | Hs.523789 |  |
| 217729_s_at | 2.1 | 2.0 | AES | amino-terminal enhancer of split | 166 | Hs.515053 | **Q08117** |
| 209203_s_at | 2.0 | 2.3 | BICD2 | bicaudal D homolog 2 (Drosophila) | 23299 | Hs.436939 | **Q8TD16** |
| 212702_s_at | 2.5 | 2.7 | BICD2 | bicaudal D homolog 2 (Drosophila) | 23299 | Hs.436939 | **Q8TD16** |
| 213154_s_at | 2.8 | 2.7 | BICD2 | bicaudal D homolog 2 (Drosophila) | 23299 | Hs.436939 | **Q8TD16** |
| 225970_at | 2.1 | 2.1 | DDHD1 | DDHD domain containing 1 | 80821 | Hs.513260 | Q8NEL9 |
| 201681_s_at | 2.8 | 2.1 | DLG5 | discs, large homolog 5 (Drosophila) | 9231 | Hs.500245 | **Q8TDM6** |
| 229689_s_at | 2.3 | 2.3 | DLG5 | discs, large homolog 5 (Drosophila) | 9231 | Hs.500245 | **Q8TDM6** |
| 204646_at | 4.6 | 2.1 | DPYD | dihydropyrimidine dehydrogenase | 1806 | Hs.335034 | **Q12882** |
| 226811_at | 2.5 | 2.1 | FAM46C | family with sequence similarity 46, member C | 54855 | Hs.356216 | **Q5VWP2** |
| 212288_at | 2.0 | 2.1 | FNBP1 | formin binding protein 1 | 23048 | Hs.189409 | **Q96RU3** |
| 209911_x_at | 2.0 | 2.2 | HIST1H2BD | histone cluster 1, H2bd | 3017 | Hs.591797 | **P58876** |
| 202202_s_at | 4.0 | 2.4 | LAMA4 | laminin, alpha 4 | 3910 | Hs.213861 | **Q16363** |
| 227940_at | 2.0 | 2.2 | LOC339803 | hypothetical protein LOC339803 | 339803 | Hs.252433 |  |
| 223852_s_at | 2.0 | 2.3 | LSM10 | LSM10, U7 small nuclear RNA associated | 84967 | Hs.471768 | **Q969L4** |
| 224675_at | 2.3 | 2.0 | MESDC2 | mesoderm development candidate 2 | 23184 | Hs.578450 | **Q14696** |
| 212473_s_at | 3.1 | 2.3 | MICAL2 | microtubule associated monoxygenase, calponin and LIM domain containing 2 | 9645 | Hs.501928 | **O94851** |
| 218678_at | 2.8 | 2.0 | NES | nestin | 10763 | Hs.527971 | **P48681** |
| 226103_at | 3.3 | 2.1 | NEXN | nexilin (F actin binding protein) | 91624 | Hs.632387 | **Q0ZGT2** |
| 212522_at | 2.2 | 2.1 | PDE8A | phosphodiesterase 8A | 5151 | Hs.9333 | O60658 |
| 206157_at | 2.6 | 2.2 | PTX3 | pentraxin-related gene, rapidly induced by IL-1 beta | 5806 | Hs.591286 | **P26022** |
| 205037_at | 2.3 | 2.2 | RABL4 | RAB, member of RAS oncogene family-like 4 | 11020 | Hs.415172 | **Q9BW83** |
| 223852_s_at | 2.0 | 2.3 | STK40 | serine/threonine kinase 40 | 83931 | Hs.471768 | **Q8N2I9** |
| 212112_s_at | 2.0 | 2.1 | STX12 | syntaxin 12 | 23673 | Hs.523855 | **Q86Y82** |
| 224593_at | 2.3 | 2.4 | ZNF664 | zinc finger protein 664 | 144348 | Hs.524828 | Q3ZCQ7 |
| 226562_at | 2.6 | 2.1 | ZNF690 | zinc finger protein 690 | 146050 | Hs.418287 | Q8IWY8 |

Probe sets listed are altered at least 2-fold downregulated (green) or upregulated (red) compared with the non-specific siRNA experiment in control cells, and were mapped to UniGene, Entrez Gene and SwissProt. Genes/proteins that were matched to protein-protein interactions (PPIs ) in I^2^D ver. 1.7 appear in boldface in SwissProt ID.

Supplementary Table 2B. List of downregulated and upregulated genes unique to cyclin D1 knockdown

| Probe set | Fold change | Gene Symbol | Gene Name | Entrez Gene | UniGene | SwissProt |
| --- | --- | --- | --- | --- | --- | --- |
| 225707_at | 0.4 | ARL6IP6 | ADP-ribosylation-like factor 6 interacting protein 6 | 151188 | Hs.516468 | **Q7Z4G7** |
| 225711_at | 0.4 | ARL6IP6 | ADP-ribosylation-like factor 6 interacting protein 6 | 151188 | Hs.516468 | **Q7Z4G7** |
| 213026_at | 0.4 | ATG12 | ATG12 autophagy related 12 homolog (S. cerevisiae) | 9140 | Hs.264482 | **O94817** |
| 226785_at | 0.5 | ATP11C | ATPase, Class VI, type 11C | 286410 | Hs.88252 | Q8NB49 |
| 223652_at | 0.5 | C10orf32 | chromosome 10 open reading frame 32 | 119032 | Hs.34492 | Q96B45 |
| 230364_at | 0.5 | CHPT1 | choline phosphotransferase 1 | 56994 | Hs.293077 | Q8WUD6 |
| 225009_at | 0.4 | CMTM4 | CKLF-like MARVEL transmembrane domain containing 4 | 146223 | Hs.643961 | Q8IZR5 |
| 222476_at | 0.4 | CNOT6 | CCR4-NOT transcription complex, subunit 6 | 57472 | Hs.157606 | **Q9ULM6** |
| 204172_at | 0.4 | CPOX | coproporphyrinogen oxidase | 1371 | Hs.476982 | **P36551** |
| 230426_at | 0.5 | DLD | dihydrolipoamide dehydrogenase | 1738 | Hs.131711 | **P09622** |
| 228033_at | 0.5 | E2F7 | E2F transcription factor 7 | 144455 | Hs.416375 | **Q96AV8** |
| 201017_at | 0.5 | EIF1AX | eukaryotic translation initiation factor 1A, X-linked | 1964 | Hs.522590 | **O75642** |
| 223068_at | 0.5 | EML4 | echinoderm microtubule associated protein like 4 | 27436 | Hs.593614 | **Q9HC35** |
| 231576_at | 0.5 | ETNK1 | ethanolamine kinase 1 | 55500 | Hs.29464 | **Q9HBU6** |
| 226965_at | 0.5 | FAM116A | family with sequence similarity 116, member A | 201627 | Hs.91085 | Q8TEG8 |
| 235030_at | 0.4 | FAM55C | family with sequence similarity 55, member C | 91775 | Hs.130195 | Q969Y0 |
| 235169_at | 0.4 | FBXO27 | F-box protein 27 | 126433 | Hs.187461 | Q8NI29 |
| 210950_s_at | 0.5 | FDFT1 | farnesyl-diphosphate farnesyltransferase 1 | 2222 | Hs.593928 | **P37268** |
| 209864_at | 0.5 | FRAT2 | frequently rearranged in advanced T-cell lymphomas 2 | 23401 | Hs.140720 | **O75474** |
| 222587_s_at | 0.4 | GALNT7 | UDP-N-acetyl-alpha-D-galactosamine:polypeptide N-acetylgalactosaminyltransferase 7 (GalNAc-T7) | 51809 | Hs.127407 | Q86SF2 |
| 218313_s_at | 0.4 | GALNT7 | UDP-N-acetyl-alpha-D-galactosamine:polypeptide N-acetylgalactosaminyltransferase 7 (GalNAc-T7) | 51809 | Hs.127407 | Q86SF2 |
| 230278_at | 0.3 | GPR137B | G protein-coupled receptor 137B | 7107 | Hs.498160 | O60478 |
| 210148_at | 0.3 | HIPK3 | homeodomain interacting protein kinase 3 | 10114 | Hs.201918 | **Q9H422** |
| 229667_s_at | 0.4 | HOXB8 | homeobox B8 | 3218 | Hs.514292 | **P17481** |
| 218091_at | 0.5 | HRB | HIV-1 Rev binding protein | 3267 | Hs.591619 | **P52594** |
| 208935_s_at | 0.5 | LGALS8 | lectin, galactoside-binding, soluble, 8 (galectin 8) | 3964 | Hs.4082 | **O00214** |
| 229349_at | 0.4 | LIN28B | lin-28 homolog B (C. elegans) | 389421 | Hs.23616 | Q6ZN17 |
| 1559139_at | 0.5 | LOC205251 | LOC205251 | 205251 | Hs.128499 | Q8NCU8 |
| 238506_at | 0.4 | LRRC58 | leucine rich repeat containing 58 | 116064 | Hs.518084 | **Q96CX6** |
| 225479_at | 0.5 | LRRC58 | leucine rich repeat containing 58 | 116064 | Hs.518084 | **Q96CX6** |
| 224568_x_at | 0.3 | MALAT1 | metastasis associated lung adenocarcinoma transcript 1 (non-coding RNA) | 378938 | Hs.642877 |  |
| 223940_x_at | 0.4 | MALAT1 | metastasis associated lung adenocarcinoma transcript 1 (non-coding RNA) | 378938 | Hs.642877 |  |
| 1558678_s_at | 0.4 | MALAT1 | metastasis associated lung adenocarcinoma transcript 1 (non-coding RNA) | 378938 | Hs.642877 |  |
| 224567_x_at | 0.4 | MALAT1 | metastasis associated lung adenocarcinoma transcript 1 (non-coding RNA) | 378938 | Hs.642877 |  |
| 227510_x_at | 0.5 | MALAT1 | metastasis associated lung adenocarcinoma transcript 1 (non-coding RNA) | 378938 | Hs.642877 |  |
| 226675_s_at | 0.5 | MALAT1 | metastasis associated lung adenocarcinoma transcript 1 (non-coding RNA) | 378938 | Hs.642877 |  |
| 231735_s_at | 0.5 | MALAT1 | metastasis associated lung adenocarcinoma transcript 1 (non-coding RNA) | 378938 | Hs.642877 |  |
| 211071_s_at | 0.4 | MLLT11 | myeloid/lymphoid or mixed-lineage leukemia (trithorax homolog, Drosophila); translocated to, 11 | 10962 | Hs.75823 | Q13015 |
| 214078_at | 0.5 | PAK3 | p21 (CDKN1A)-activated kinase 3 | 5063 | Hs.390616 | **O75914** |
| 213372_at | 0.4 | PAQR3 | progestin and adipoQ receptor family member III | 152559 | Hs.632591 | Q6TCH7 |
| 229830_at | 0.4 | PDGFA | platelet-derived growth factor alpha polypeptide | 5154 | Hs.645488 | **P04085** |
| 225829_at | 0.3 | PDZD8 | PDZ domain containing 8 | 118987 | Hs.501149 | Q8NEN9 |
| 225367_at | 0.4 | PGM2 | phosphoglucomutase 2 | 55276 | Hs.23363 | **Q96G03** |
| 223738_s_at | 0.5 | PGM2 | phosphoglucomutase 2 | 55276 | Hs.23363 | **Q96G03** |
| 228722_at | 0.4 | PRMT2 | protein arginine methyltransferase 2 | 3275 | Hs.154163 | **P55345** |
| 218053_at | 0.4 | PRPF40A | PRP40 pre-mRNA processing factor 40 homolog A (yeast) | 55660 | Hs.591637 | **O75400** |
| 233080_s_at | 0.5 | PRPF40A | PRP40 pre-mRNA processing factor 40 homolog A (yeast) | 55660 | Hs.591637 | **O75400** |
| 225796_at | 0.5 | PXK | PX domain containing serine/threonine kinase | 54899 | Hs.190544 | Q7Z7A4 |
| 228030_at | 0.4 | RBM6 | RNA binding motif protein 6 | 10180 | Hs.596224 | P78332 |
| 218793_s_at | 0.4 | SCML1 | sex comb on midleg-like 1 (Drosophila) | 6322 | Hs.109655 | Q9UN30 |
| 228026_at | 0.3 | SIKE | suppressor of IKK epsilon | 80143 | Hs.632428 | Q5TEZ9 |
| 204666_s_at | 0.4 | SIKE | suppressor of IKK epsilon | 80143 | Hs.632428 | Q5TEZ9 |
| 221705_s_at | 0.4 | SIKE | suppressor of IKK epsilon | 80143 | Hs.632428 | Q5TEZ9 |
| 212281_s_at | 0.5 | TMEM97 | transmembrane protein 97 | 27346 | Hs.199695 | Q5BJF2 |
| 205347_s_at | 0.5 | TMSL8 | thymosin-like 8 | 11013 | Hs.56145 | Q99406 |
| 222480_at | 0.5 | UBE2Q1 | ubiquitin-conjugating enzyme E2Q (putative) 1 | 55585 | Hs.607928 | Q7Z7E8 |
| 235003_at | 0.3 | UHMK1 | U2AF homology motif (UHM) kinase 1 | 127933 | Hs.127310 | **Q8TAS1** |
| 1552656_s_at | 0.4 | UHMK1 | U2AF homology motif (UHM) kinase 1 | 127933 | Hs.127310 | **Q8TAS1** |
| 212388_at | 0.2 | USP24 | ubiquitin specific peptidase 24 | 23358 | Hs.477009 | Q9UPU5 |
| 238057_at | 0.5 | USP45 | ubiquitin specific peptidase 45 | 85015 | Hs.143410 | **Q70EL2** |
| 1557132_at | 0.4 | WDR17 | WD repeat domain 17 | 116966 | Hs.532056 | Q8IZU2 |
| 203505_at | 2.2 | ABCA1 | ATP-binding cassette, sub-family A (ABC1), member 1 | 19 | Hs.429294 | **O95477** |
| 209765_at | 2.1 | ADAM19 | ADAM metallopeptidase domain 19 (meltrin beta) | 8728 | Hs.483944 | **Q9H013** |
| 204497_at | 2.4 | ADCY9 | adenylate cyclase 9 | 115 | Hs.391860 | O60503 |
| 230275_at | 2.4 | ARSI | arylsulfatase family, member I | 340075 | Hs.591252 | Q5FYB1 |
| 215037_s_at | 2.1 | BCL2L1 | BCL2-like 1 | 598 | Hs.516966 | **Q07817** |
| 228434_at | 2.2 | BTNL9 | butyrophilin-like 9 | 153579 | Hs.546502 | Q6UXG8 |
| 226487_at | 2.1 | C12orf34 | chromosome 12 open reading frame 34 | 84915 | Hs.144655 | Q5U5X8 |
| 219563_at | 2.3 | C14orf139 | chromosome 14 open reading frame 139 | 79686 | Hs.41502 | **Q9H761** |
| 232235_at | 2.6 | C18orf4 | chromosome 18 open reading frame 4 | 92126 | Hs.124673 | Q8IZU8 |
| 243292_at | 2.9 | C1QDC2 | C1q domain containing 2 | 388581 | Hs.197613 | Q5T7M4 |
| 225048_at | 2.1 | C6orf120 | chromosome 6 open reading frame 120 | 387263 | Hs.435933 | Q7Z4R8 |
| 224370_s_at | 2.1 | CAPS2 | calcyphosine 2 | 84698 | Hs.407154 | Q9BXY5 |
| 207181_s_at | 2.1 | CASP7 | caspase 7, apoptosis-related cysteine peptidase | 840 | Hs.9216 | **P55210** |
| 211922_s_at | 2.3 | CAT | catalase | 847 | Hs.502302 | **P04040** |
| 201432_at | 2.1 | CAT | catalase | 847 | Hs.502302 | **P04040** |
| 201925_s_at | 2.2 | CD55 | CD55 molecule, decay accelerating factor for complement (Cromer blood group) | 1604 | Hs.527653 | **P08174** |
| 228748_at | 2.5 | CD59 | CD59 molecule, complement regulatory protein | 966 | Hs.278573 | **P13987** |
| 209288_s_at | 2.3 | CDC42EP3 | CDC42 effector protein (Rho GTPase binding) 3 | 10602 | Hs.369574 | **Q9UKI2** |
| 209286_at | 2.1 | CDC42EP3 | CDC42 effector protein (Rho GTPase binding) 3 | 10602 | Hs.369574 | **Q9UKI2** |
| 235443_at | 2.7 | CKMT2 | creatine kinase, mitochondrial 2 (sarcomeric) | 1160 | Hs.80691 | **P17540** |
| 218182_s_at | 2.3 | CLDN1 | claudin 1 | 9076 | Hs.439060 | **O95832** |
| 229271_x_at | 3.6 | COL11A1 | collagen, type XI, alpha 1 | 1301 | Hs.523446 | **P12107** |
| 37892_at | 2.8 | COL11A1 | collagen, type XI, alpha 1 | 1301 | Hs.523446 | **P12107** |
| 204320_at | 2.2 | COL11A1 | collagen, type XI, alpha 1 | 1301 | Hs.523446 | **P12107** |
| 206100_at | 3.0 | CPM | carboxypeptidase M | 1368 | Hs.484551 | **P14384** |
| 227473_at | 2.1 | CTTN | cortactin | 2017 | Hs.632133 | **Q14247** |
| 214743_at | 2.1 | CUTL1 | cut-like 1, CCAAT displacement protein (Drosophila) | 1523 | Hs.191482 | **P39880** |
| 234915_s_at | 2.3 | DENR | density-regulated protein | 8562 | Hs.22393 | **O43583** |
| 221509_at | 2.2 | DENR | density-regulated protein | 8562 | Hs.22393 | **O43583** |
| 231896_s_at | 2.1 | DENR | density-regulated protein | 8562 | Hs.22393 | **O43583** |
| 218995_s_at | 2.1 | EDN1 | endothelin 1 | 1906 | Hs.621232 | **P05305** |
| 204797_s_at | 2.2 | EML1 | echinoderm microtubule associated protein like 1 | 2009 | Hs.12451 | **O00423** |
| 238533_at | 2.0 | EPHA7 | EPH receptor A7 | 2045 | Hs.73962 | **Q15375** |
| 201889_at | 2.6 | FAM3C | family with sequence similarity 3, member C | 10447 | Hs.434053 | Q92520 |
| 225864_at | 3.3 | FAM84B | family with sequence similarity 84, member B | 157638 | Hs.124951 | **Q96KN1** |
| 204359_at | 2.1 | FLRT2 | fibronectin leucine rich transmembrane protein 2 | 23768 | Hs.533710 | **O43155** |
| 225481_at | 2.3 | FRMD6 | FERM domain containing 6 | 122786 | Hs.434914 | **Q96NE9** |
| 225464_at | 2.1 | FRMD6 | FERM domain containing 6 | 122786 | Hs.434914 | **Q96NE9** |
| 229441_at | 2.0 | FZD4 | frizzled homolog 4 (Drosophila) | 8322 | Hs.591968 | **Q9ULV1** |
| 208841_s_at | 2.3 | G3BP2 | GTPase activating protein (SH3 domain) binding protein 2 | 9908 | Hs.303676 | **Q9UN86** |
| 223278_at | 3.9 | GJB2 | gap junction protein, beta 2, 26kDa (connexin 26) | 2706 | Hs.591234 | **P29033** |
| 231771_at | 3.0 | GJB6 | gap junction protein, beta 6 (connexin 30) | 10804 | Hs.511757 | **O95452** |
| 221869_at | 2.4 | GM632 | KIAA1196 protein | 57473 | Hs.551552 | **Q96KM6** |
| 224646_x_at | 2.2 | H19 | H19, imprinted maternally expressed untranslated mRNA | 283120 | Hs.533566 |  |
| 223541_at | 2.6 | HAS3 | hyaluronan synthase 3 | 3038 | Hs.592069 | O00219 |
| 202815_s_at | 2.1 | HEXIM1 | hexamethylene bis-acetamide inducible 1 | 10614 | Hs.15299 | **O94992** |
| 211538_s_at | 2.4 | HSPA2 | heat shock 70kDa protein 2 | 3306 | Hs.432648 | **P54652** |
| 201466_s_at | 2.4 | JUN | jun oncogene | 3725 | Hs.525704 | **P05412** |
| 224392_s_at | 3.8 | KMO | kynurenine 3-monooxygenase (kynurenine 3-hydroxylase) | 8564 | Hs.409081 | **O15229** |
| 219032_x_at | 3.5 | KMO | kynurenine 3-monooxygenase (kynurenine 3-hydroxylase) | 8564 | Hs.409081 | **O15229** |
| 204734_at | 3.0 | KRT15 | keratin 15 | 3866 | Hs.2785 | **P19012** |
| 235497_at | 2.0 | LOC643837 | hypothetical protein LOC643837 | 643837 | Hs.593676 | Q96BN7 |
| 225540_at | 3.1 | LOC648149 | hypothetical protein LOC648149 | 648149 | Hs.368281 | Q6ZWB4 |
| 225540_at | 3.1 | MAP2 | microtubule-associated protein 2 | 4133 | Hs.368281 | **P11137** |
| 218181_s_at | 2.2 | MAP4K4 | mitogen-activated protein kinase kinase kinase kinase 4 | 9448 | Hs.431550 | **O95819** |
| 226487_at | 2.1 | MGC14436 | hypothetical protein MGC14436 | 84983 | Hs.144655 | Q96I53 |
| 206100_at | 3.0 | MGC5370 | hypothetical protein MGC5370 | 84825 | Hs.484551 |  |
| 211026_s_at | 2.7 | MGLL | monoglyceride lipase | 11343 | Hs.277035 | **Q99685** |
| 225782_at | 2.0 | MSRB3 | methionine sulfoxide reductase B3 | 253827 | Hs.339024 | **Q6UXS2** |
| 214156_at | 2.0 | MYRIP | myosin VIIA and Rab interacting protein | 25924 | Hs.594535 | **Q8NFW9** |
| 229461_x_at | 8.8 | NEGR1 | neuronal growth regulator 1 | 257194 | Hs.146542 | **Q7Z3B1** |
| 243357_at | 2.3 | NEGR1 | neuronal growth regulator 1 | 257194 | Hs.146542 | **Q7Z3B1** |
| 242539_at | 2.0 | NRBF2 | nuclear receptor binding factor 2 | 29982 | Hs.449628 | **Q96F24** |
| 224392_s_at | 3.8 | OPN3 | opsin 3 (encephalopsin, panopsin) | 23596 | Hs.409081 | Q9H1Y3 |
| 219032_x_at | 3.5 | OPN3 | opsin 3 (encephalopsin, panopsin) | 23596 | Hs.409081 | Q9H1Y3 |
| 232054_at | 2.8 | PCDH20 | protocadherin 20 | 64881 | Hs.391781 | Q8N6Y1 |
| 229256_at | 2.8 | PGM2L1 | phosphoglucomutase 2-like 1 | 283209 | Hs.26612 | Q6PCE3 |
| 229553_at | 2.4 | PGM2L1 | phosphoglucomutase 2-like 1 | 283209 | Hs.26612 | Q6PCE3 |
| 238417_at | 2.4 | PGM2L1 | phosphoglucomutase 2-like 1 | 283209 | Hs.26612 | Q6PCE3 |
| 225048_at | 2.1 | PHF10 | PHD finger protein 10 | 55274 | Hs.435933 | **Q8WUB8** |
| 221538_s_at | 2.7 | PLXNA1 | plexin A1 | 5361 | Hs.432329 | **Q9UIW2** |
| 201578_at | 3.3 | PODXL | podocalyxin-like | 5420 | Hs.16426 | **O00592** |
| 217944_at | 2.0 | POMGNT1 | protein O-linked mannose beta1,2-N-acetylglucosaminyltransferase | 55624 | Hs.525134 | Q8WZA1 |
| 209529_at | 2.1 | PPAP2C | phosphatidic acid phosphatase type 2C | 8612 | Hs.465506 | **O43688** |
| 203355_s_at | 2.3 | PSD3 | pleckstrin and Sec7 domain containing 3 | 23362 | Hs.434255 | **Q9NYI0** |
| 210355_at | 2.2 | PTHLH | parathyroid hormone-like hormone | 5744 | Hs.591159 | **P12272** |
| 208300_at | 2.1 | PTPRH | protein tyrosine phosphatase, receptor type, H | 5794 | Hs.179770 | **Q15426** |
| 238447_at | 2.3 | RBMS3 | RNA binding motif, single stranded interacting protein | 27303 | Hs.696468 | Q6XE24 |
| 202388_at | 2.8 | RGS2 | regulator of G-protein signalling 2, 24kDa | 5997 | Hs.78944 | **P41220** |
| 201204_s_at | 2.2 | RRBP1 | ribosome binding protein 1 homolog 180kDa (dog) | 6238 | Hs.472213 | **Q9P2E9** |
| 202627_s_at | 2.3 | SERPINE1 | serpin peptidase inhibitor, clade E (nexin, plasminogen activator inhibitor type 1), member 1 | 5054 | Hs.414795 | **P05121** |
| 201739_at | 2.3 | SGK | serum/glucocorticoid regulated kinase | 6446 | Hs.510078 | **O00141** |
| 235050_at | 2.1 | SLC2A12 | solute carrier family 2 (facilitated glucose transporter), member 12 | 154091 | Hs.486508 | Q8TD20 |
| 232481_s_at | 2.5 | SLITRK6 | SLIT and NTRK-like family, member 6 | 84189 | Hs.525105 | Q9H5Y7 |
| 212956_at | 2.3 | TBC1D9 | TBC1 domain family, member 9 (with GRAM domain) | 23158 | Hs.480819 | O94958 |
| 201042_at | 2.0 | TGM2 | transglutaminase 2 (C polypeptide, protein-glutamine-gamma-glutamyltransferase) | 7052 | Hs.517033 | **P21980** |
| 201110_s_at | 5.2 | THBS1 | thrombospondin 1 | 7057 | Hs.164226 | **P07996** |
| 201109_s_at | 3.4 | THBS1 | thrombospondin 1 | 7057 | Hs.164226 | **P07996** |
| 211689_s_at | 2.8 | TMPRSS2 | transmembrane protease, serine 2 | 7113 | Hs.439309 | **O15393** |
| 210986_s_at | 2.1 | TPM1 | tropomyosin 1 (alpha) | 7168 | Hs.133892 | **P09493** |
| 210987_x_at | 2.1 | TPM1 | tropomyosin 1 (alpha) | 7168 | Hs.133892 | **P09493** |
| 221571_at | 2.3 | TRAF3 | TNF receptor-associated factor 3 | 7187 | Hs.510528 | **Q13114** |
| 215111_s_at | 2.1 | TSC22D1 | TSC22 domain family, member 1 | 8848 | Hs.507916 | **Q15714** |
| 55872_at | 2.6 | UCKL1 | uridine-cytidine kinase 1-like 1 | 54963 | Hs.504998 | **Q9NWZ5** |
| 211009_s_at | 2.1 | ZNF271 | zinc finger protein 271 | 10778 | Hs.314246 | Q14591 |
| 206557_at | 2.7 | ZNF320 | zinc finger protein 320 | 162967 | Hs.467223 | Q6ZP55 |
| 214751_at | 2.2 | ZNF320 | zinc finger protein 320 | 162967 | Hs.467223 | Q6ZP55 |
| 206557_at | 2.7 | ZNF468 | zinc finger protein 468 | 90333 | Hs.467223 | Q5CZB8 |
| 214751_at | 2.2 | ZNF468 | zinc finger protein 468 | 90333 | Hs.467223 | Q5CZB8 |
| 204453_at | 2.1 | ZNF84 | zinc finger protein 84 | 7637 | Hs.445019 | **P51523** |

Probe sets listed are altered at least 2-fold downregulated (green) or upregulated (red) compared to the non-specific siRNA experiment in control cells, and were mapped to UniGene, Entrez Gene and SwissProt. Genes/proteins that were matched to protein-protein interactions (PPIs) in I^2^D ver. 1.7 appear in boldface in SwissProt ID.

Supplementary Table 2C. List of downregulated and upregulated genes unique to cyclin D3 knockdown

| Probe set | Fold change | Gene Symbol | Gene Name | Entrez Gene | UniGene | SwissProt |
| --- | --- | --- | --- | --- | --- | --- |
| 1552287_s_at | 0.5 | AFG3L1 | AFG3 ATPase family gene 3-like 1 (S. cerevisiae) | 172 | Hs.534773 | O43931 |
| 221505_at | 0.5 | ANP32E | acidic (leucine-rich) nuclear phosphoprotein 32 family, member E | 81611 | Hs.603000 | Q9BTT0 |
| 229128_s_at | 0.5 | ANP32E | acidic (leucine-rich) nuclear phosphoprotein 32 family, member E | 81611 | Hs.603000 | Q9BTT0 |
| 200761_s_at | 0.5 | ARL6IP5 | ADP-ribosylation-like factor 6 interacting protein 5 | 10550 | Hs.518060 | **O75915** |
| 203487_s_at | 0.5 | ARMC8 | armadillo repeat containing 8 | 25852 | Hs.266826 | **Q6PIL2** |
| 226517_at | 0.5 | BCAT1 | branched chain aminotransferase 1, cytosolic | 586 | Hs.438993 | **P54687** |
| 224367_at | 0.5 | BEX2 | brain expressed X-linked 2 | 84707 | Hs.398989 | **Q9BXY8** |
| 224719_s_at | 0.5 | C12orf57 | chromosome 12 open reading frame 57 | 113246 | Hs.591045 | Q99622 |
| 218940_at | 0.4 | C14orf138 | chromosome 14 open reading frame 138 | 79609 | Hs.558541 | Q9H867 |
| 239038_at | 0.4 | C1orf52 | chromosome 1 open reading frame 52 | 148423 | Hs.26226 | Q8N6N3 |
| 227627_at | 0.5 | C8orf44 | chromosome 8 open reading frame 44 | 56260 | Hs.545401 | Q96CB5 |
| 202284_s_at | 0.5 | CDKN1A | cyclin-dependent kinase inhibitor 1A (p21, Cip1) | 1026 | Hs.370771 | **P38936** |
| 205474_at | 0.4 | CRLF3 | cytokine receptor-like factor 3 | 51379 | Hs.370168 | **Q8IUI8** |
| 201372_s_at | 0.5 | CUL3 | cullin 3 | 8452 | Hs.372286 | **Q13618** |
| 219825_at | 0.4 | CYP26B1 | cytochrome P450, family 26, subfamily B, polypeptide 1 | 56603 | Hs.91546 | Q9NR63 |
| 204244_s_at | 0.4 | DBF4 | DBF4 homolog (S. cerevisiae) | 10926 | Hs.485380 | **Q9UBU7** |
| 218277_s_at | 0.4 | DHX40 | DEAH (Asp-Glu-Ala-His) box polypeptide 40 | 79665 | Hs.29403 | Q8IX18 |
| 224215_s_at | 0.5 | DLL1 | delta-like 1 (Drosophila) | 28514 | Hs.379912 | **O00548** |
| 222692_s_at | 0.5 | FNDC3B | fibronectin type III domain containing 3B | 64778 | Hs.159430 | Q53EP0 |
| 222693_at | 0.4 | FNDC3B | fibronectin type III domain containing 3B | 64778 | Hs.159430 | Q53EP0 |
| 211458_s_at | 0.5 | GABARAPL3 | GABA(A) receptors associated protein like 3 | 23766 | Hs.592014 | **Q9BY60** |
| 207574_s_at | 0.4 | GADD45B | growth arrest and DNA-damage-inducible, beta | 4616 | Hs.110571 | **O75293** |
| 209304_x_at | 0.5 | GADD45B | growth arrest and DNA-damage-inducible, beta | 4616 | Hs.110571 | **O75293** |
| 231871_at | 0.4 | GPR180 | G protein-coupled receptor 180 | 160897 | Hs.439363 | Q86V85 |
| 203632_s_at | 0.5 | GPRC5B | G protein-coupled receptor, family C, group 5, member B | 51704 | Hs.148685 | Q9NZH0 |
| 207002_s_at | 0.5 | HYMAI | hydatidiform mole associated and imprinted | 57061 | Hs.444975 |  |
| 219209_at | 0.5 | IFIH1 | interferon induced with helicase C domain 1 | 64135 | Hs.163173 | **Q9BYX4** |
| 226757_at | 0.5 | IFIT2 | interferon-induced protein with tetratricopeptide repeats 2 | 3433 | Hs.437609 | **P09913** |
| 225511_at | 0.5 | IQCK | IQ motif containing K | 124152 | Hs.460217 | Q8N0W5 |
| 219479_at | 0.5 | KDELC1 | KDEL (Lys-Asp-Glu-Leu) containing 1 | 79070 | Hs.408629 | Q6UW63 |
| 223800_s_at | 0.4 | LIMS3 | LIM and senescent cell antigen-like domains 3 | 96626 | Hs.535619 | Q9HB10 |
| 231871_at | 0.4 | LOC144874 | hypothetical protein LOC144874 | 144874 | Hs.439363 |  |
| 223800_s_at | 0.4 | LOC440895 | similar to LIM and senescent cell antigen-like domains 3 | 440895 | Hs.535619 |  |
| 228423_at | 0.5 | MAP9 | microtubule-associated protein 9 | 79884 | Hs.61271 | Q49MG5 |
| 227379_at | 0.5 | MBOAT1 | membrane bound O-acyltransferase domain containing 1 | 154141 | Hs.377830 | Q6ZNC8 |
| 202731_at | 0.5 | PDCD4 | programmed cell death 4 (neoplastic transformation inhibitor) | 27250 | Hs.232543 | **Q53EL6** |
| 212593_s_at | 0.4 | PDCD4 | programmed cell death 4 (neoplastic transformation inhibitor) | 27250 | Hs.232543 | **Q53EL6** |
| 207002_s_at | 0.5 | PLAGL1 | pleiomorphic adenoma gene-like 1 | 5325 | Hs.444975 | **Q9UM63** |
| 201375_s_at | 0.4 | PPP2CB | protein phosphatase 2 (formerly 2A), catalytic subunit, beta isoform | 5516 | Hs.491440 | **P62714** |
| 227627_at | 0.5 | PTTG3 | pituitary tumor-transforming 3 | 26255 | Hs.545401 | O95356 |
| 209515_s_at | 0.4 | RAB27A | RAB27A, member RAS oncogene family | 5873 | Hs.493512 | **P51159** |
| 227369_at | 0.5 | SERBP1 | SERPINE1 mRNA binding protein 1 | 26135 | Hs.530412 | **Q8NC51** |
| 227627_at | 0.5 | SGK3 | serum/glucocorticoid regulated kinase family, member 3 | 23678 | Hs.545401 | **Q96BR1** |
| 210567_s_at | 0.5 | SKP2 | S-phase kinase-associated protein 2 (p45) | 6502 | Hs.23348 | **Q13309** |
| 229065_at | 0.4 | SLC35F3 | solute carrier family 35, member F3 | 148641 | Hs.158748 | Q8N9C9 |
| 1555460_a_at | 0.5 | SLC39A6 | solute carrier family 39 (zinc transporter), member 6 | 25800 | Hs.79136 | **Q13433** |
| 202089_s_at | 0.4 | SLC39A6 | solute carrier family 39 (zinc transporter), member 6 | 25800 | Hs.79136 | **Q13433** |
| 209198_s_at | 0.4 | SYT11 | synaptotagmin XI | 23208 | Hs.32984 | **Q9BT88** |
| 201448_at | 0.5 | TIA1 | TIA1 cytotoxic granule-associated RNA binding protein | 7072 | Hs.516075 | **P31483** |
| 227459_at | 0.4 | TMEM140 | transmembrane protein 140 | 55281 | Hs.567530 | Q8WUC3 |
| 228029_at | 2.2 | ABCA11 | ATP-binding cassette, sub-family A (ABC1), member 11 (pseudogene) | 79963 | Hs.428360 | Q4W5N1 |
| 201284_s_at | 2.0 | APEH | N-acylaminoacyl-peptide hydrolase | 327 | Hs.517969 | **P13798** |
| 212423_at | 2.1 | C10orf56 | chromosome 10 open reading frame 56 | 219654 | Hs.523080 | Q5U5T9 |
| 233543_s_at | 2.1 | CCDC98 | coiled-coil domain containing 98 | 84142 | Hs.334772 | **Q6UWZ7** |
| 235287_at | 2.0 | CDK6 | cyclin-dependent kinase 6 | 1021 | Hs.119882 | **Q00534** |
| 211934_x_at | 2.2 | GANAB | glucosidase, alpha; neutral AB | 23193 | Hs.595071 | **Q14697** |
| 214626_s_at | 2.2 | GANAB | glucosidase, alpha; neutral AB | 23193 | Hs.595071 | **Q14697** |
| 212355_at | 2.2 | KIAA0323 | KIAA0323 | 23351 | Hs.643552 | **O15037** |
| 228029_at | 2.2 | LOC152719 | hypothetical protein LOC152719 | 152719 | Hs.428360 |  |
| 238437_at | 2.1 | LOC390980 | similar to Zinc finger protein 264 | 390980 | Hs.22488 | Q5CZA5 |
| 207098_s_at | 2.0 | MFN1 | mitofusin 1 | 55669 | Hs.478383 | **Q8IWA4** |
| 232028_at | 2.0 | MGC15634 | hypothetical protein MGC15634 | 84841 | Hs.30323 |  |
| 218951_s_at | 2.1 | PLCXD1 | phosphatidylinositol-specific phospholipase C, X domain containing 1 | 55344 | Hs.522568 | Q9NUJ7 |
| 212561_at | 2.1 | RAB6IP1 | RAB6 interacting protein 1 | 23258 | Hs.501857 | **Q6IQ26** |
| 205485_at | 2.2 | RYR1 | ryanodine receptor 1 (skeletal) | 6261 | Hs.466664 | **P21817** |
| 224928_at | 2.3 | SETD7 | SET domain containing (lysine methyltransferase) 7 | 80854 | Hs.480792 | **Q8WTS6** |
| 210664_s_at | 2.1 | TFPI | tissue factor pathway inhibitor (lipoprotein-associated coagulation inhibitor) | 7035 | Hs.516578 | **P10646** |
| 221255_s_at | 2.0 | TMEM93 | transmembrane protein 93 | 83460 | Hs.30011 | **Q9BV81** |
| 244551_at | 2.0 | TRIM60 | tripartite motif-containing 60 | 166655 | Hs.368004 | Q495X7 |
| 210276_s_at | 2.5 | TRIOBP | TRIO and F-actin binding protein | 11078 | Hs.533030 | **Q9H2D6** |
| 216210_x_at | 2.1 | TRIOBP | TRIO and F-actin binding protein | 11078 | Hs.533030 | **Q9H2D6** |
| 203597_s_at | 2.1 | WBP4 | WW domain binding protein 4 (formin binding protein 21) | 11193 | Hs.411300 | **O75554** |
| 219540_at | 2.4 | ZNF267 | zinc finger protein 267 | 10308 | Hs.460645 | Q14586 |
| 1558722_at | 2.2 | ZNF454 | zinc finger protein 454 | 285676 | Hs.259441 | Q8N9F8 |
| 232028_at | 2.0 | ZNF678 | zinc finger protein 678 | 339500 | Hs.30323 | Q5SXM1 |
| 229533_x_at | 2.0 | ZNF680 | zinc finger protein 680 | 340252 | Hs.520886 | Q8NEM1 |
| 239482_x_at | 2.1 | ZNF708 | zinc finger protein 708 | 7562 | Hs.466296 | P17019 |
| 228029_at | 2.2 | ZNF721 | zinc finger protein 721 | 170960 | Hs.428360 | Q8TF20 |
| 206059_at | 2.0 | ZNF91 | zinc finger protein 91 | 7644 | Hs.631626 | **Q05481** |

Probe sets listed are altered at least 2-fold downregulated (green) or upregulated (red) compared to the non-specific siRNA experiment in control cells, and were mapped to UniGene, Entrez Gene and SwissProt. Genes/proteins that were matched to protein-protein interactions (PPIs) in I^2^D ver. 1.7 appear in boldface in SwissProt ID.
