# Supplementary material for: Differential roles of cyclin D1 and D3 in pancreatic ductal adenocarcinoma
Source: Mol Cancer. 2010 Feb 1;9:24. doi: 10.1186/1476-4598-9-24 (PMC2824633; doi:10.1186/1476-4598-9-24)
Supplement: Additional file 7 — Supplementary Table 4A-4D. Functional annotation of deregulated common target genes in D1- or D3-cyclin suppressed cells and their interacting proteins obtained from protein interaction database I2D ver. 1.7.. Significant associations with GO biological processes are shown for common deregulated genes in either D1 or D3 cyclin siRNA-treated cells. Listed are terms where values of enrichment >2 and p-value < 0.05. Enrichment and FDR are as per GoMiner analysis (GO database version 2007-06) described in Materials and Methods. [file 1476-4598-9-24-S7.DOC]

Supplementary Table 4A Functional annotation of deregulated common target genes in D1- or D3-cyclin suppressed cells and their interacting proteins obtained from protein interaction database I^2^D ver. 1.7.

| **GO Category** | **Total Genes** | **Number of deregulated genes** | **Enrichment** | **FDR (p-value)** |
| --- | --- | --- | --- | --- |
| GO:0016055-Wnt receptor signaling pathway | 87 | 13 | 4.99 | 0.0000 |
| GO:0006944-membrane fusion | 25 | 7 | 9.35 | 0.0013 |
| GO:0051649-establishment of cellular localization | 624 | 39 | 2.09 | 0.0015 |
| GO:0048489-synaptic vesicle transport | 16 | 6 | 12.53 | 0.002 |
| GO:0051641-cellular localization | 637 | 39 | 2.05 | 0.0026 |
| GO:0046907-intracellular transport | 514 | 33 | 2.14 | 0.0033 |
| GO:0016192-vesicle-mediated transport | 359 | 26 | 2.42 | 0.0033 |
| GO:0016044-membrane organization and biogenesis | 206 | 18 | 2.92 | 0.004 |
| GO:0015031-protein transport | 504 | 32 | 2.12 | 0.0043 |
| GO:0008104-protein localization | 565 | 34 | 2.01 | 0.0064 |
| GO:0006412-translation | 341 | 24 | 2.35 | 0.0076 |
| GO:0006886-intracellular protein transport | 295 | 21 | 2.38 | 0.0078 |
| GO:0030111-regulation of Wnt receptor signaling pathway | 19 | 5 | 8.79 | 0.0081 |
| GO:0001933-negative regulation of protein amino acid phosphorylation | 5 | 3 | 20.04 | 0.0097 |
| GO:0045763-negative regulation of amino acid metabolic process | 5 | 3 | 20.04 | 0.0097 |
| GO:0042326-negative regulation of phosphorylation | 6 | 3 | 16.7 | 0.0157 |
| GO:0045936-negative regulation of phosphate metabolic process | 6 | 3 | 16.7 | 0.0157 |
| GO:0006521-regulation of amino acid metabolic process | 37 | 6 | 5.42 | 0.0169 |
| GO:0018108-peptidyl-tyrosine phosphorylation | 37 | 6 | 5.42 | 0.0169 |
| GO:0007268-synaptic transmission | 213 | 16 | 2.51 | 0.0172 |
| GO:0001932-regulation of protein amino acid phosphorylation | 36 | 6 | 5.57 | 0.0175 |
| GO:0006414-translational elongation | 14 | 4 | 9.54 | 0.0179 |
| GO:0018212-peptidyl-tyrosine modification | 38 | 6 | 5.27 | 0.0181 |
| GO:0007260-tyrosine phosphorylation of STAT protein | 15 | 4 | 8.91 | 0.0184 |
| GO:0016079-synaptic vesicle exocytosis | 7 | 3 | 14.32 | 0.0204 |
| GO:0042516-regulation of tyrosine phosphorylation of Stat3 protein | 7 | 3 | 14.32 | 0.0204 |
| GO:0010033-response to organic substance | 27 | 5 | 6.19 | 0.0211 |
| GO:0050730-regulation of peptidyl-tyrosine phosphorylation | 27 | 5 | 6.19 | 0.0211 |
| GO:0006904-vesicle docking during exocytosis | 16 | 4 | 8.35 | 0.0219 |
| GO:0006470-protein amino acid dephosphorylation | 107 | 10 | 3.12 | 0.0252 |
| GO:0042503-tyrosine phosphorylation of Stat3 protein | 8 | 3 | 12.53 | 0.0256 |
| GO:0022406-membrane docking | 17 | 4 | 7.86 | 0.0259 |
| GO:0048278-vesicle docking | 17 | 4 | 7.86 | 0.0259 |
| GO:0006261-DNA-dependent DNA replication | 76 | 8 | 3.52 | 0.0301 |
| GO:0042733-embryonic digit morphogenesis | 9 | 3 | 11.14 | 0.0331 |
| GO:0019226-transmission of nerve impulse | 239 | 16 | 2.24 | 0.035 |
| GO:0042325-regulation of phosphorylation | 46 | 6 | 4.36 | 0.035 |
| GO:0006260-DNA replication | 156 | 12 | 2.57 | 0.0356 |
| GO:0007215-glutamate signaling pathway | 20 | 4 | 6.68 | 0.0365 |
| GO:0042509-regulation of tyrosine phosphorylation of STAT protein | 10 | 3 | 10.02 | 0.0384 |
| GO:0046425-regulation of JAK-STAT cascade | 10 | 3 | 10.02 | 0.0384 |
| GO:0016311-dephosphorylation | 121 | 10 | 2.76 | 0.0404 |
| GO:0019220-regulation of phosphate metabolic process | 49 | 6 | 4.09 | 0.0405 |
| GO:0051174-regulation of phosphorus metabolic process | 49 | 6 | 4.09 | 0.0405 |
| GO:0045045-secretory pathway | 182 | 13 | 2.39 | 0.0411 |
| GO:0006903-vesicle targeting | 11 | 3 | 9.11 | 0.0443 |
| GO:0007004-telomere maintenance via telomerase | 11 | 3 | 9.11 | 0.0443 |
| GO:0031324-negative regulation of cellular metabolic process | 276 | 17 | 2.06 | 0.0454 |
| GO:0006302-double-strand break repair | 22 | 4 | 6.07 | 0.0459 |
| GO:0051325-interphase | 69 | 7 | 3.39 | 0.0477 |
| GO:0051329-interphase of mitotic cell cycle | 69 | 7 | 3.39 | 0.0477 |
| GO:0017038-protein import | 87 | 8 | 3.07 | 0.0482 |

Significant associations with GO biological processes are shown for common deregulated genes in either D1 or D3 cyclin siRNA-treated cells. Listed are terms where values of enrichment >2 and p-value <0.05. Enrichment and FDR are as per GoMiner analysis (GO database version 2007-06) described in Materials and Methods.

Supplementary Table 4B Functional annotation of down-regulated target genes in D3-cyclin suppressed cells.

| **GO Category** | **Total Genes** | **Number of deregulated genes** | **Enrichment** | **FDR (p-value)** |
| --- | --- | --- | --- | --- |
| GO:0000082-G1 S transition of mitotic cell cycle | 28 | 4 | 61.46 | 0.0000 |
| GO:0022402-cell cycle process | 584 | 9 | 6.63 | 0.0015 |
| GO:0012501-programmed cell death | 573 | 8 | 6.01 | 0.0033 |
| GO:0007049-cell cycle | 680 | 9 | 5.69 | 0.0037 |
| GO:0012502-induction of programmed cell death | 160 | 5 | 13.45 | 0.0038 |
| GO:0006917-induction of apoptosis | 159 | 5 | 13.53 | 0.0039 |
| GO:0000074-regulation of progression through cell cycle | 446 | 7 | 6.75 | 0.0041 |
| GO:0008219-cell death | 609 | 8 | 5.65 | 0.0045 |
| GO:0016265-death | 609 | 8 | 5.65 | 0.0045 |
| GO:0006915-apoptosis | 568 | 8 | 6.06 | 0.0045 |
| GO:0043068-positive regulation of programmed cell death | 188 | 5 | 11.44 | 0.0046 |
| GO:0051726-regulation of cell cycle | 450 | 7 | 6.69 | 0.0048 |
| GO:0051325-interphase | 69 | 4 | 24.94 | 0.0048 |
| GO:0043065-positive regulation of apoptosis | 186 | 5 | 11.57 | 0.0049 |
| GO:0006469-negative regulation of protein kinase activity | 37 | 3 | 34.89 | 0.0052 |
| GO:0051348-negative regulation of transferase activity | 37 | 3 | 34.89 | 0.0052 |
| GO:0051329-interphase of mitotic cell cycle | 68 | 4 | 25.31 | 0.0060 |
| GO:0000278-mitotic cell cycle | 222 | 5 | 9.69 | 0.0063 |
| GO:0022403-cell cycle phase | 242 | 5 | 8.89 | 0.0081 |
| GO:0043067-regulation of programmed cell death | 386 | 6 | 6.69 | 0.0085 |
| GO:0042981-regulation of apoptosis | 381 | 6 | 6.78 | 0.0089 |
| GO:0043086-negative regulation of enzyme activity | 58 | 3 | 22.25 | 0.0106 |
| GO:0007050-cell cycle arrest | 63 | 3 | 20.49 | 0.0120 |
| GO:0045786-negative regulation of progression through cell cycle | 162 | 4 | 10.62 | 0.0128 |
| GO:0048468-cell development | 898 | 8 | 3.83 | 0.0154 |
| GO:0030154-cell differentiation | 1320 | 9 | 2.93 | 0.0321 |
| GO:0048869-cellular developmental process | 1320 | 9 | 2.93 | 0.0321 |
| GO:0008629-induction of apoptosis by intracellular signals | 28 | 2 | 30.73 | 0.0344 |

Significant associations with GO biological processes are shown in the down-regulated genes in cyclin D3 siRNA-treated cells. Listed are terms where values of enrichment >2 and p-value <0.05. Enrichment and FDR are as per GoMiner analysis (GO database version 2007-06) described in Materials and Methods.

Supplementary Table 4C. Functional annotation using GO terms for deregulated target genes in cyclin D3 suppressed cells and their interacting proteins obtained from protein interaction database I^2^D ver. 1.7.

| **GO Category** | **Total Genes** | **Number of deregulated genes** | **Enrichment** | **FDR ( p-value)** |
| --- | --- | --- | --- | --- |
| GO:0006259-DNA metabolic process | 550 | 64 | 2.02 | 0.0000 |
| GO:0022403-cell cycle phase | 242 | 38 | 2.73 | 0.0000 |
| GO:0007049-cell cycle | 681 | 108 | 2.76 | 0.0000 |
| GO:0022402-cell cycle process | 586 | 94 | 2.79 | 0.0000 |
| GO:0000278-mitotic cell cycle | 223 | 36 | 2.81 | 0.0000 |
| GO:0008380-RNA splicing | 180 | 31 | 2.99 | 0.0000 |
| GO:0030163-protein catabolic process | 170 | 30 | 3.07 | 0.0000 |
| GO:0043549-regulation of kinase activity | 182 | 33 | 3.15 | 0.0000 |
| GO:0051338-regulation of transferase activity | 182 | 33 | 3.15 | 0.0000 |
| GO:0045786-negative regulation of progression through cell cycle | 158 | 29 | 3.19 | 0.0000 |
| GO:0045859-regulation of protein kinase activity | 179 | 33 | 3.20 | 0.0000 |
| GO:0000074-regulation of progression through cell cycle | 443 | 83 | 3.26 | 0.0000 |
| GO:0051726-regulation of cell cycle | 447 | 84 | 3.27 | 0.0000 |
| GO:0006260-DNA replication | 156 | 34 | 3.79 | 0.0000 |
| GO:0007050-cell cycle arrest | 61 | 18 | 5.13 | 0.0000 |
| GO:0006261-DNA-dependent DNA replication | 76 | 23 | 5.26 | 0.0000 |
| GO:0051325-interphase | 69 | 21 | 5.29 | 0.0000 |
| GO:0051329-interphase of mitotic cell cycle | 69 | 21 | 5.29 | 0.0000 |
| GO:0000079-regulation of cyclin-dependent protein kinase activity | 39 | 17 | 7.57 | 0.0000 |
| GO:0006270-DNA replication initiation | 25 | 14 | 9.73 | 0.0000 |
| GO:0006397-mRNA processing | 206 | 31 | 2.61 | 0.0001 |
| GO:0065009-regulation of a molecular function | 361 | 45 | 2.17 | 0.0001 |
| GO:0051301-cell division | 184 | 29 | 2.74 | 0.0001 |
| GO:0051052-regulation of DNA metabolic process | 40 | 12 | 5.21 | 0.0001 |
| GO:0000080-G1 phase of mitotic cell cycle | 16 | 8 | 8.69 | 0.0001 |
| GO:0051318-G1 phase | 16 | 8 | 8.69 | 0.0001 |
| GO:0006511-ubiquitin-dependent protein catabolic process | 123 | 22 | 3.11 | 0.0001 |
| GO:0043285-biopolymer catabolic process | 232 | 32 | 2.40 | 0.0001 |
| GO:0000082-G1 S transition of mitotic cell cycle | 29 | 10 | 5.99 | 0.0001 |
| GO:0044257-cellular protein catabolic process | 125 | 22 | 3.06 | 0.0001 |
| GO:0051603-proteolysis involved in cellular protein catabolic process | 125 | 22 | 3.06 | 0.0001 |
| GO:0016071-mRNA metabolic process | 241 | 32 | 2.31 | 0.0001 |
| GO:0019941-modification-dependent protein catabolic process | 124 | 22 | 3.08 | 0.0001 |
| GO:0043632-modification-dependent macromolecule catabolic process | 124 | 22 | 3.08 | 0.0001 |
| GO:0050790-regulation of catalytic activity | 342 | 41 | 2.08 | 0.0001 |
| GO:0006275-regulation of DNA replication | 20 | 8 | 6.95 | 0.0002 |
| GO:0006974-response to DNA damage stimulus | 240 | 31 | 2.24 | 0.0003 |
| GO:0008629-induction of apoptosis by intracellular signals | 28 | 9 | 5.59 | 0.0003 |
| GO:0000075-cell cycle checkpoint | 43 | 11 | 4.45 | 0.0003 |
| GO:0043065-positive regulation of apoptosis | 197 | 27 | 2.38 | 0.0003 |
| GO:0043068-positive regulation of programmed cell death | 199 | 27 | 2.36 | 0.0003 |
| GO:0000389-nuclear mRNA 3'-splice site recognition | 5 | 4 | 13.90 | 0.0008 |
| GO:0009719-response to endogenous stimulus | 268 | 32 | 2.07 | 0.0009 |
| GO:0006469-negative regulation of protein kinase activity | 40 | 10 | 4.34 | 0.0010 |
| GO:0051348-negative regulation of transferase activity | 40 | 10 | 4.34 | 0.0010 |
| GO:0007219-Notch signaling pathway | 33 | 9 | 4.74 | 0.0011 |
| GO:0043086-negative regulation of enzyme activity | 60 | 12 | 3.48 | 0.0015 |
| GO:0007089-traversing start control point of mitotic cell cycle | 6 | 4 | 11.58 | 0.0017 |
| GO:0000375-RNA splicing via transesterification reactions | 63 | 12 | 3.31 | 0.0021 |
| GO:0000377-RNA splicing via transesterification reactions with bulged adenosine as nucleophile | 63 | 12 | 3.31 | 0.0021 |
| GO:0000398-nuclear mRNA splicing via spliceosome | 63 | 12 | 3.31 | 0.0021 |
| GO:0030518-steroid hormone receptor signaling pathway | 37 | 9 | 4.23 | 0.0022 |
| GO:0006917-induction of apoptosis | 168 | 22 | 2.28 | 0.0024 |
| GO:0012502-induction of programmed cell death | 169 | 22 | 2.26 | 0.0025 |
| GO:0045595-regulation of cell differentiation | 114 | 17 | 2.59 | 0.0025 |
| GO:0030522-intracellular receptor-mediated signaling pathway | 39 | 9 | 4.01 | 0.0026 |
| GO:0030521-androgen receptor signaling pathway | 25 | 7 | 4.87 | 0.0040 |
| GO:0031570-DNA integrity checkpoint | 19 | 6 | 5.49 | 0.0045 |
| GO:0051053-negative regulation of DNA metabolic process | 13 | 5 | 6.68 | 0.0050 |
| GO:0007259-JAK-STAT cascade | 34 | 8 | 4.09 | 0.0050 |
| GO:0007215-glutamate signaling pathway | 20 | 6 | 5.21 | 0.0066 |
| GO:0006414-translational elongation | 14 | 5 | 6.21 | 0.0073 |
| GO:0045893-positive regulation of transcription DNA-dependent | 175 | 21 | 2.09 | 0.0086 |
| GO:0007346-regulation of progression through mitotic cell cycle | 23 | 6 | 4.53 | 0.0122 |
| GO:0045944-positive regulation of transcription from RNA polymerase II promoter | 100 | 14 | 2.43 | 0.0133 |
| GO:0008156-negative regulation of DNA replication | 10 | 4 | 6.95 | 0.0144 |
| GO:0000076-DNA replication checkpoint | 5 | 3 | 10.43 | 0.0171 |
| GO:0018106-peptidyl-histidine phosphorylation | 5 | 3 | 10.43 | 0.0171 |
| GO:0032297-negative regulation of DNA replication initiation | 5 | 3 | 10.43 | 0.0171 |
| GO:0006457-protein folding | 171 | 20 | 2.03 | 0.0175 |
| GO:0006266-DNA ligation | 6 | 3 | 8.69 | 0.0287 |
| GO:0007216-metabotropic glutamate receptor signaling pathway | 6 | 3 | 8.69 | 0.0287 |
| GO:0007220-Notch receptor processing | 6 | 3 | 8.69 | 0.0287 |
| GO:0000270-peptidoglycan metabolic process | 19 | 5 | 4.57 | 0.0301 |
| GO:0000086-G2 M transition of mitotic cell cycle | 13 | 4 | 5.35 | 0.0368 |
| GO:0006376-mRNA splice site selection | 13 | 4 | 5.35 | 0.0368 |
| GO:0018202-peptidyl-histidine modification | 7 | 3 | 7.45 | 0.0425 |
| GO:0030174-regulation of DNA replication initiation | 7 | 3 | 7.45 | 0.0425 |
| GO:0045736-negative regulation of cyclin-dependent protein kinase activity | 7 | 3 | 7.45 | 0.0425 |

Significant associations with GO biological processes are shown for common deregulated genes in cyclin D3 siRNA-treated cells. Listed are terms where values of enrichment >2 and p-value <0.05. Enrichment and FDR are as per GoMiner analysis (GO database version 2007-06) described in Materials and Methods.

Supplementary Table 4D. Functional annotation using GO terms for deregulated target genes in cyclin D1 suppressed cells and their interacting proteins obtained from protein interaction databse I^2^D ver. 1.7.

| **GO Category** | **Total Genes** | **Number of deregulated genes** | **Enrichment** | **FDR (p-value)** |
| --- | --- | --- | --- | --- |
| GO:0008219-cell death | 635 | 181 | 2.2 | 0.0000 |
| GO:0016265-death | 635 | 181 | 2.2 | 0.0000 |
| GO:0012501-programmed cell death | 603 | 174 | 2.2 | 0.0000 |
| GO:0006915-apoptosis | 598 | 172 | 2.2 | 0.0000 |
| GO:0043067-regulation of programmed cell death | 420 | 133 | 2.4 | 0.0000 |
| GO:0042981-regulation of apoptosis | 415 | 131 | 2.4 | 0.0000 |
| GO:0008632-apoptotic program | 71 | 35 | 3.7 | 0.0000 |
| GO:0043069-negative regulation of programmed cell death | 186 | 62 | 2.5 | 0.0000 |
| GO:0043066-negative regulation of apoptosis | 184 | 61 | 2.5 | 0.0000 |
| GO:0006916-anti-apoptosis | 146 | 51 | 2.6 | 0.0000 |
| GO:0043068-positive regulation of programmed cell death | 199 | 60 | 2.3 | 0.0000 |
| GO:0043065-positive regulation of apoptosis | 197 | 59 | 2.3 | 0.0000 |
| GO:0006917-induction of apoptosis | 168 | 49 | 2.2 | 0.0000 |
| GO:0012502-induction of programmed cell death | 169 | 49 | 2.2 | 0.0000 |
| GO:0030029-actin filament-based process | 172 | 47 | 2.1 | 0.0000 |
| GO:0030036-actin cytoskeleton organization and biogenesis | 161 | 44 | 2.1 | 0.0000 |
| GO:0006914-autophagy | 16 | 10 | 4.7 | 0.0001 |
| GO:0001836-release of cytochrome c from mitochondria | 11 | 8 | 5.5 | 0.0002 |
| GO:0008637-apoptotic mitochondrial changes | 17 | 10 | 4.4 | 0.0002 |
| GO:0006919-caspase activation | 31 | 14 | 3.4 | 0.0002 |
| GO:0006606-protein import into nucleus | 72 | 23 | 2.4 | 0.0004 |
| GO:0007249-I-kappaB kinase NF-kappaB cascade | 108 | 30 | 2.1 | 0.0005 |
| GO:0046902-regulation of mitochondrial membrane permeability | 5 | 5 | 7.6 | 0.0005 |
| GO:0043281-regulation of caspase activity | 42 | 16 | 2.9 | 0.0006 |
| GO:0051170-nuclear import | 74 | 23 | 2.3 | 0.0007 |
| GO:0051223-regulation of protein transport | 23 | 11 | 3.6 | 0.0009 |
| GO:0043280-positive regulation of caspase activity | 35 | 14 | 3.0 | 0.0010 |
| GO:0051098-regulation of binding | 24 | 11 | 3.5 | 0.0012 |
| GO:0043123-positive regulation of I-kappaB kinase NF-kappaB cascade | 69 | 21 | 2.3 | 0.0020 |
| GO:0030518-steroid hormone receptor signaling pathway | 37 | 14 | 2.9 | 0.0020 |
| GO:0043122-regulation of I-kappaB kinase NF-kappaB cascade | 75 | 22 | 2.2 | 0.0021 |
| GO:0007005-mitochondrion organization and biogenesis | 38 | 14 | 2.8 | 0.0022 |
| GO:0008634-negative regulation of survival gene product activity | 6 | 5 | 6.3 | 0.0026 |
| GO:0032386-regulation of intracellular transport | 22 | 10 | 3.4 | 0.0028 |
| GO:0007596-blood coagulation | 82 | 23 | 2.1 | 0.0031 |
| GO:0018193-peptidyl-amino acid modification | 82 | 23 | 2.1 | 0.0031 |
| GO:0030522-intracellular receptor-mediated signaling pathway | 39 | 14 | 2.7 | 0.0032 |
| GO:0017038-protein import | 87 | 24 | 2.1 | 0.0032 |
| GO:0006921-cell structure disassembly during apoptosis | 19 | 9 | 3.6 | 0.0036 |
| GO:0050817-coagulation | 83 | 23 | 2.1 | 0.0038 |
| GO:0032446-protein modification by small protein conjugation | 61 | 18 | 2.2 | 0.0059 |
| GO:0007599-hemostasis | 86 | 23 | 2.0 | 0.0060 |
| GO:0008635-caspase activation via cytochrome c | 7 | 5 | 5.4 | 0.0070 |
| GO:0030521-androgen receptor signaling pathway | 25 | 10 | 3.0 | 0.0079 |
| GO:0046822-regulation of nucleocytoplasmic transport | 25 | 10 | 3.0 | 0.0079 |
| GO:0051345-positive regulation of hydrolase activity | 58 | 17 | 2.2 | 0.0088 |
| GO:0007015-actin filament organization | 30 | 11 | 2.8 | 0.0089 |
| GO:0000389-nuclear mRNA 3'-splice site recognition | 5 | 4 | 6.0 | 0.0141 |
| GO:0018105-peptidyl-serine phosphorylation | 8 | 5 | 4.7 | 0.0161 |
| GO:0018209-peptidyl-serine modification | 8 | 5 | 4.7 | 0.0161 |
| GO:0006611-protein export from nucleus | 15 | 7 | 3.5 | 0.0166 |
| GO:0033157-regulation of intracellular protein transport | 19 | 8 | 3.2 | 0.0170 |
| GO:0042306-regulation of protein import into nucleus | 19 | 8 | 3.2 | 0.0170 |
| GO:0001942-hair follicle development | 12 | 6 | 3.8 | 0.0182 |
| GO:0022404-molting cycle process | 12 | 6 | 3.8 | 0.0182 |
| GO:0022405-hair cycle process | 12 | 6 | 3.8 | 0.0182 |
| GO:0042303-molting cycle | 12 | 6 | 3.8 | 0.0182 |
| GO:0042345-regulation of NF-kappaB import into nucleus | 12 | 6 | 3.8 | 0.0182 |
| GO:0042348-NF-kappaB import into nucleus | 12 | 6 | 3.8 | 0.0182 |
| GO:0042633-hair cycle | 12 | 6 | 3.8 | 0.0182 |
| GO:0016567-protein ubiquitination | 57 | 16 | 2.1 | 0.0188 |
| GO:0030262-apoptotic nuclear changes | 20 | 8 | 3.0 | 0.0194 |
| GO:0051402-neuron apoptosis | 20 | 8 | 3.0 | 0.0194 |
| GO:0007006-mitochondrial membrane organization and biogenesis | 16 | 7 | 3.3 | 0.0202 |
| GO:0030330-DNA damage response signal transduction by p53 class mediator | 9 | 5 | 4.2 | 0.0236 |
| GO:0006084-acetyl-CoA metabolic process | 25 | 9 | 2.7 | 0.0244 |
| GO:0051259-protein oligomerization | 44 | 13 | 2.2 | 0.0249 |
| GO:0000045-autophagic vacuole formation | 6 | 4 | 5.0 | 0.0274 |
| GO:0006266-DNA ligation | 6 | 4 | 5.0 | 0.0274 |
| GO:0051235-maintenance of localization | 30 | 10 | 2.5 | 0.0281 |
| GO:0050818-regulation of coagulation | 13 | 6 | 3.5 | 0.0292 |
| GO:0050819-negative regulation of coagulation | 13 | 6 | 3.5 | 0.0292 |
| GO:0022411-cellular component disassembly | 35 | 11 | 2.4 | 0.0305 |
| GO:0051260-protein homooligomerization | 26 | 9 | 2.6 | 0.0317 |
| GO:0051051-negative regulation of transport | 22 | 8 | 2.7 | 0.0338 |
| GO:0009952-anterior posterior pattern formation | 46 | 13 | 2.1 | 0.0343 |
| GO:0007016-cytoskeletal anchoring | 10 | 5 | 3.8 | 0.0363 |
| GO:0031365-N-terminal protein amino acid modification | 10 | 5 | 3.8 | 0.0363 |
| GO:0045580-regulation of T cell differentiation | 10 | 5 | 3.8 | 0.0363 |
| GO:0051101-regulation of DNA binding | 10 | 5 | 3.8 | 0.0363 |
| GO:0000060-protein import into nucleus translocation | 18 | 7 | 2.9 | 0.0367 |
| GO:0006891-intra-Golgi vesicle-mediated transport | 18 | 7 | 2.9 | 0.0367 |
| GO:0042990-regulation of transcription factor import into nucleus | 18 | 7 | 2.9 | 0.0367 |
| GO:0042991-transcription factor import into nucleus | 18 | 7 | 2.9 | 0.0367 |
| GO:0065002-intracellular protein transport across a membrane | 18 | 7 | 2.9 | 0.0367 |
| GO:0045884-regulation of survival gene product activity | 14 | 6 | 3.2 | 0.0387 |
| GO:0002367-cytokine production during immune response | 7 | 4 | 4.3 | 0.0454 |
| GO:0002700-regulation of production of molecular mediator of immune response | 7 | 4 | 4.3 | 0.0454 |
| GO:0002718-regulation of cytokine production during immune response | 7 | 4 | 4.3 | 0.0454 |
| GO:0016236-macroautophagy | 7 | 4 | 4.3 | 0.0454 |
| GO:0030032-lamellipodium biogenesis | 7 | 4 | 4.3 | 0.0454 |
| GO:0051881-regulation of mitochondrial membrane potential | 7 | 4 | 4.3 | 0.0454 |
| GO:0008629-induction of apoptosis by intracellular signals | 28 | 9 | 2.4 | 0.0463 |
| GO:0006413-translational initiation | 43 | 12 | 2.1 | 0.0466 |

Significant associations with GO biological processes are shown for the common deregulated genes in cyclin D1 siRNA-treated cells. Listed are terms where values of enrichment >2 and p-value <0.05. Enrichment and FDR are as per GoMiner analysis (GO database version 2007-06) described in Materials and Methods.
