# Supplementary material for: Differential roles of cyclin D1 and D3 in pancreatic ductal adenocarcinoma
Source: Mol Cancer. 2010 Feb 1;9:24. doi: 10.1186/1476-4598-9-24 (PMC2824633; doi:10.1186/1476-4598-9-24)
Supplement: Additional file 8 — Supplementary Table 5. KEGG functional annotation of deregulated target genes in D1- or D3-cyclin suppressed cells and their interacting proteins obtained from protein interaction database I2D ver. 1.7. ([23]; http://ophid.utoronto.ca/i2d). Listed are number of genes mapped to a selection of 38 cancer and signaling KEGG pathways; common target genes are downregulated (down) or upregulated (up) by either D1- or D3-cyclin siRNA treatment (D1/D3). Following are deregulated genes (down or up) unique to either cyclin D1 (D1) or cyclin D3 (D3) siRNA treatment. A significant enrichment (red highlighted) for KEGG pathway cell cycle proteins was found for cyclin D3 downregulated gene targets (p = 0.0048). PPI network analysis showed significant enrichments (red highlighted) of two KEGG pathways: p53 signaling (p = 0.0008) and cell cycle (p = 0.004). [file 1476-4598-9-24-S8.DOC]

Supplementary Table 5. KEGG functional annotation of deregulated target genes in D1- or D3-cyclin suppressed cells and their interacting proteins obtained from protein interaction database I^2^D ver. 1.7.

| **KEGG pathway title** | **KEGG ID** | **Genes down both in D1/D3** | **Genes up both in D1/D3** | **Genes down in D1** | **Genes up in D1** | **Genes down in D3** | **Genes up in D3** | **PPI_up/ down both in D1/D3** | **PPI_up/ down in D1** | **PPI_up/ down in D3** |
| --- | --- | --- | --- | --- | --- | --- | --- | --- | --- | --- |
| all genes |  | 12 | 22 | 47 | 84 | 45 | 27 |  |  |  |
| mapped to ppi networks |  | 5 | 17 | 24 | 57 | 20 | 12 | 289 | 1362 | 576 |
| genes mapped to KEGG |  | 1 | 1 | 3 | 13 | 6 | 2 | 56 | 313 | 168 |
| Regulation of actin cytoskeleton | 4810 | 0 | 0 | 2 | 0 | 0 | 0 | 6 | 53 | 11 |
| Adherens junction | 4520 | 0 | 0 | 0 | 0 | 0 | 0 | 6 | 35 | 13 |
| Acute myeloid leukemia | 5221 | 0 | 0 | 0 | 0 | 0 | 0 | 3 | 18 | 18 |
| Apoptosis | 4210 | 0 | 0 | 0 | 2 | 0 | 0 | 8 | 40 | 9 |
| Basal cell carcinoma | 5217 | 0 | 0 | 0 | 1 | 0 | 0 | 2 | 7 | 7 |
| Calcium signaling pathway | 4020 | 0 | 0 | 0 | 1 | 0 | 1 | 8 | 20 | 16 |
| Cell adhesion molecules (CAMs) | 4514 | 0 | 0 | 0 | 2 | 0 | 0 | 0 | 14 | 3 |
| Cell cycle | 4110 | 0 | 0 | 0 | 0 | 4 | 1 | 9 | 24 | 53 |
| Chronic myeloid leukemia | 5220 | 0 | 0 | 0 | 1 | 1 | 1 | 8 | 26 | 21 |
| Colorectal cancer | 5210 | 0 | 0 | 0 | 2 | 0 | 0 | 6 | 37 | 20 |
| Cytokine-cytokine receptor interaction | 4060 | 0 | 0 | 0 | 0 | 0 | 0 | 5 | 31 | 6 |
| ECM-receptor interaction | 4512 | 0 | 1 | 0 | 2 | 0 | 0 | 1 | 20 | 1 |
| Endometrial cancer | 5213 | 0 | 0 | 0 | 0 | 0 | 0 | 3 | 21 | 13 |
| ErbB signaling pathway | 4012 | 0 | 0 | 1 | 1 | 1 | 0 | 1 | 27 | 18 |
| Focal adhesion | 4510 | 0 | 1 | 2 | 3 | 0 | 0 | 6 | 67 | 20 |
| Gap junction | 4540 | 0 | 0 | 1 | 1 | 0 | 0 | 5 | 24 | 9 |
| Glioma | 5214 | 0 | 0 | 1 | 0 | 1 | 1 | 5 | 21 | 17 |
| Hedgehog signaling pathway | 4340 | 0 | 0 | 0 | 0 | 0 | 0 | 2 | 4 | 5 |
| Insulin signaling pathway | 4910 | 0 | 0 | 0 | 0 | 0 | 0 | 7 | 29 | 18 |
| Jak-STAT signaling pathway | 4630 | 0 | 0 | 0 | 1 | 0 | 0 | 2 | 16 | 17 |
| MAPK signaling pathway | 4010 | 0 | 0 | 1 | 2 | 1 | 0 | 13 | 62 | 26 |
| Melanoma | 5218 | 0 | 0 | 1 | 0 | 1 | 1 | 6 | 18 | 11 |
| mTOR signaling pathway | 4150 | 0 | 0 | 0 | 0 | 0 | 0 | 3 | 8 | 9 |
| Non-small cell lung cancer | 5223 | 0 | 0 | 0 | 0 | 0 | 1 | 4 | 22 | 14 |
| Notch signaling pathway | 4330 | 0 | 0 | 0 | 0 | 1 | 0 | 2 | 10 | 9 |
| p53 signaling pathway | 4115 | 0 | 0 | 0 | 2 | 2 | 1 | 4 | 18 | 23 |
| Pancreatic cancer | 5212 | 0 | 0 | 0 | 1 | 0 | 1 | 10 | 33 | 18 |
| Phosphatidylinositol signaling system | 4070 | 0 | 0 | 0 | 0 | 0 | 0 | 0 | 8 | 5 |
| PPAR signaling pathway | 3320 | 0 | 0 | 0 | 0 | 0 | 0 | 0 | 8 | 6 |
| Prostate cancer | 5215 | 0 | 0 | 1 | 0 | 1 | 0 | 7 | 35 | 22 |
| Renal cell carcinoma | 5211 | 0 | 0 | 1 | 1 | 0 | 0 | 2 | 24 | 8 |
| Small cell lung cancer | 5222 | 0 | 1 | 0 | 2 | 1 | 1 | 6 | 36 | 20 |
| TGF-beta signaling pathway | 4350 | 1 | 0 | 0 | 1 | 1 | 0 | 9 | 20 | 19 |
| Thyroid cancer | 5216 | 0 | 0 | 0 | 0 | 0 | 0 | 2 | 8 | 9 |
| Tight junction | 4530 | 1 | 0 | 0 | 2 | 1 | 0 | 13 | 30 | 13 |
| Toll-like receptor signaling pathway | 4620 | 0 | 0 | 0 | 1 | 0 | 0 | 3 | 25 | 6 |
| VEGF signaling pathway | 4370 | 0 | 0 | 0 | 0 | 0 | 0 | 3 | 21 | 6 |
| Wnt signaling pathway | 4310 | 1 | 0 | 1 | 2 | 1 | 0 | 15 | 34 | 33 |

Listed are number of genes mapped to a selection of 38 cancer and signaling KEGG pathways; common target genes are downregulated (down) or upregulated (up) by either D1- or D3-cyclin siRNA treatment (D1/D3). Following are deregulated genes (down or up) unique to either cyclin D1 (D1) or cyclin D3 (D3) siRNA treatment. A significant enrichment (red highlighted) for KEGG pathway cell cycle proteins was found for cyclin D3 downregulated gene targets (p=0.0048). PPI network analysis showed significant enrichments (red highlighted) of two KEGG pathways: p53 signaling (p=0.0008) and cell cycle (p=0.004).
