# Supplementary material for: Differential roles of cyclin D1 and D3 in pancreatic ductal adenocarcinoma
Source: Mol Cancer. 2010 Feb 1;9:24. doi: 10.1186/1476-4598-9-24 (PMC2824633; doi:10.1186/1476-4598-9-24)
Supplement: Additional file 9 — Supplementary Figure 4. Quantification of western blots in Figure 6. Values are means ± SEM of at least three relative intensities compared to untreated control in each blot. An asterisk represents statistically significant value calculated by Student t-test (P < 0.01). [file 1476-4598-9-24-S9.DOC]

Supplementary Figure 4. Quantification of western blots in Figure 6

|  |  |  |  |  |  |  |
| --- | --- | --- | --- | --- | --- | --- |
| **Antibody** | ***BxPC3+UO126*** | ***HPAC+UO126*** | ***PANC+UO126*** | ***BxPC3+WM*** | ***HPAC+WM*** | ***PANC+WM*** |
| CCND1 | 13.48+1.231* | 45.26+26.38 | 27.50+10.8 | 79.35+21.57 | 129.7+37.93 | 106.9+36.09 |
| CCND3 | 103.2+24.45 | 26.59+7.007* | 61.99+11.36 | 86.47+10.91 | 83.86+18.17* | 74.20+6.912 |
|  |  |  |  |  |  |  |

Values are means+SEM of at least three Relative Intensities compared to untreated control in each blot. * represents statistically significant value calculated by Student t test (P<0.01).
